# Kaposi’s Sarcoma-Associated Herpesvirus Drives a Super-Enhancer-Mediated Survival Gene Expression Program in Primary Effusion Lymphoma

**DOI:** 10.1128/mBio.01457-20

**Published:** 2020-08-25

**Authors:** Mark Manzano, Thomas Günther, Hyunwoo Ju, John Nicholas, Elizabeth T. Bartom, Adam Grundhoff, Eva Gottwein

**Affiliations:** a Department of Microbiology-Immunology, Northwestern University, Chicago, Illinois, USA; b Heinrich Pette Institute, Leibniz Institute for Experimental Virology, Hamburg, Germany; c Department of Oncology, Johns Hopkins University School of Medicine, Baltimore, Maryland, USA; d Department of Biochemistry and Molecular Genetics, Northwestern University, Chicago, Illinois, USA; University of North Carolina, Chapel Hill

**Keywords:** EBNA3C, EBV, HBZ, HTLV-1, IRF4, KSHV, LANA2, master transcription factor, primary effusion lymphoma, super-enhancer, transcriptional reprogramming, vIRF3

## Abstract

Kaposi’s sarcoma-associated herpesvirus (KSHV) causes primary effusion lymphoma (PEL). The cellular transcription factor (TF) interferon (IFN) regulatory factor 4 (IRF4) is an essential oncogene in PEL, but its specific role in PEL and how KSHV deregulates IRF4 remain unknown. Here, we report that the KSHV latency protein viral interferon regulatory factor 3 (vIRF3) cooperates with IRF4 and cellular BATF (basic leucine zipper ATF-like TF) to drive a super-enhancer (SE)-mediated oncogenic transcriptional program in PEL. Chromatin immunoprecipitation coupled with next-generation sequencing (ChIP-Seq) experiments demonstrated that IRF4, vIRF3, and BATF cooccupy the SEs of key survival genes, in a pattern that is distinct from those seen with other IRF4-driven malignancies. All three proteins cooperatively drive SE-mediated IRF4 overexpression. Inactivation of vIRF3 and, to a lesser extent, BATF phenocopies the gene expression changes and loss of cellular viability observed upon inactivation of IRF4. In sum, this work suggests that KSHV vIRF3 and cellular IRF4 and BATF cooperate as oncogenic transcription factors on SEs to promote cellular survival and proliferation in KSHV-associated lymphomas.

## Abstract (precis)

Kaposi’s sarcoma-associated herpesvirus (KSHV) causes the aggressive disease primary effusion lymphoma (PEL). Here, we show that a viral transcription factor (vIRF3) cooperates with the cellular transcription factor IRF4 to control an oncogenic gene expression program in PEL cells. These proteins promote KSHV-mediated B cell transformation by activating the expression of prosurvival genes through super-enhancers. Our report thus demonstrates that this DNA tumor virus encodes a transcription factor that functions with cellular IRF4 to drive oncogenic transcriptional reprogramming.

## INTRODUCTION

The human oncogenic gammaherpesvirus Kaposi’s sarcoma-associated herpesvirus (KSHV) causes Kaposi’s sarcoma, B cell primary effusion lymphoma (PEL), and the lymphoproliferative disorder multicentric Castleman’s disease (1–4). PEL has a particularly poor outcome, with a median survival of less than 2 years (5–8). The lack of effective treatment options for PEL reflects the aggressiveness of PEL but also our poor understanding of how KSHV induces the tumor cell survival and proliferation that are hallmarks of PEL and other cancers (9). KSHV infection of all tumor cells is the defining feature of PEL. Approximately 80% of PEL tumors additionally carry Epstein-Barr virus (EBV; reviewed in reference 10). The vast majority of the PEL tumor cells exhibit a restricted latent KSHV gene expression program, including viral microRNAs, latency-associated nuclear antigen (LANA), and viral homologs of cyclin D2 (vCYC), FLICE-inhibitory protein (vFLIP), and viral interferon regulatory factors (vIRFs), including vIRF3. PEL-derived cell lines require at least KSHV LANA, vFLIP, and vIRF3 for survival and proliferation, independently of their EBV infection status (11–13). Therefore, latent KSHV infection is an invariant driver of PEL lymphomagenesis and of maintenance of all PEL-derived cell lines. EBV gene expression in the EBV-positive (EBV^+^) subset of PEL cell lines is highly restricted, with expression of only EBV nuclear antigen 1 (EBNA1) and various noncoding RNAs and of low levels of latent membrane protein 2A (LMP2A; 14). A role for EBV in PEL lymphomagenesis and in maintenance of EBV-positive PEL cell lines is suggested by studies in mouse models and in PEL cell lines (15–18). Finally, it is likely that various cellular mutations can contribute to PEL, especially in EBV-negative cases, since mutations of tumor suppressor genes *RB1*, *TP53*, and *PTEN* are present in a subset of PEL cell lines (3, 19, 20).

We recently employed genome-wide CRISPR knockout (KO) screens in a panel of eight PEL cell lines to define which human genes are required for survival and proliferation of PEL cells (21). A comparison of our results with similar data from 15 other cancer types identified 210 PEL-specific cellular oncogenic dependencies (PSODs). PSODs are genes that are essential in most or all PEL cell lines but are unlikely to be required for the survival of all other cancer cell types. These analyses revealed a uniform dependency of PEL cells on the lymphoid transcription factor (TF) interferon regulatory factor 4 (IRF4). IRF4 is a master regulator of B cell development, where it drives plasma cell differentiation. IRF4 is emerging as an oncogene involved in a subset of hematological malignancies, including multiple myeloma (MM [22]), the activated B cell-like subtype of diffuse large B cell lymphoma (ABC-DLBCL [23, 24]), anaplastic large cell lymphoma (25, 26), and human T cell leukemia virus type 1 (HTLV-1)-associated adult T cell leukemia/lymphoma (ATLL [27, 28]). Interestingly, IRF4 is also essential in lymphoblastoid cell lines (LCLs [29, 30]), which result from *in vitro* transformation of B cells by EBV. In these other settings, IRF4 binds to enhancers, including tightly clustered enhancers referred to as “super-enhancers” (SEs), to drive overexpression of MYC or, in ATLL, the essential IRF4 cotranscription factor BATF3 (basic leucine zipper ATF-like TF 3 [27, 31–34]). We and others have confirmed experimentally that PEL-derived cell lines are highly dependent on IRF4 expression (21, 35, 36). IRF4 also drives overexpression of MYC in PEL (35), by unknown mechanisms. Although IRF4 addiction is a critical feature of PEL cell lines, little is known about IRF4 regulation and function in PEL other than its role in the expression of MYC. IRF4 addiction potentially presents an attractive therapeutic opportunity in PEL, since IRF4 can likely be targeted by immunomodulatory drugs (IMiDs) (35–37). Any role of IRF4 in SE-dependent transcriptional networks in PEL could potentially also be targeted by bromodomain and extraterminal (BET) protein inhibitors (26, 27, 32), which disrupt SE function. While BET inhibitors have known high toxicity in PEL cells (35, 38), the identity and regulation of SEs in PEL cells have not been studied.

Here, we show that IRF4, the KSHV-encoded B cell-specific transcription factor vIRF3, and the cellular transcription factor BATF (basic leucine zipper ATF-like TF) exhibit overlapping genome occupancies of super-enhancer elements in PEL cells. Functional experiments and transcriptome profiling after IRF4, BATF, or vIRF3 inhibition strongly suggest that these transcription factors cooperate on super-enhancers to drive the expression of many essential genes and PSODs and thereby promote the survival and proliferation of these virally transformed B cells.

## RESULTS

### The IRF4 cofactor BATF is essential in PEL cell lines and promotes IRF4 expression.

**FIG 1:**
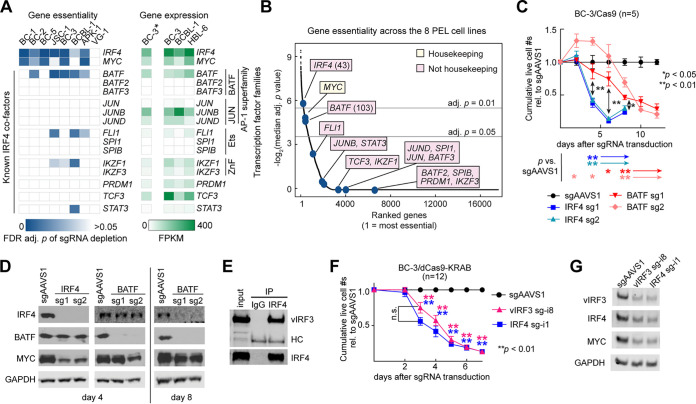
Cellular BATF and KSHV vIRF3 are candidates for essential cofactors and regulators of IRF4. (A to D) BATF, a cellular co-TF of IRF4 in other settings, is essential across PEL cell lines. (A) (Left) Blue heat map showing essentiality in eight PEL cell lines of genes that are cofactors of IRF4 in other settings. The heat map depicts false-discovery-rate (FDR)-adj. *P* values of depletion of the sgRNAs targeting these genes in our recent PEL CRISPR/Cas9 gene essentiality screens (21). VG-1 cells performed relatively poorly in these screens, but we have previously shown that IRF4 is similarly essential in VG-1 (36). While BATF did not meet our essentiality cutoff (for example, in BC-1) in our 14-day screens, BATF essentiality in BC-1 was confirmed (see Fig. S3). (Right) Green heat map showing expression (in fragments per kilobase [of transcript] per million mapped reads, FPKM) of the same genes in a BC3 RNA-Seq data set generated in this study (marked by an asterisk; see below) and published RNA-Seq data (42). (B) All genes screened previously by Manzano et al. (21) were ranked by their median FDR-adj. *P* values of sgRNA depletion (negative log_2_-transformed) across the 8 PEL cell lines. Both IRF4 and BATF scored among the most essential genes across the eight PEL cell lines. Yellow indicates that MYC scored as essential in all 16 cancer cell types analyzed in this study and is therefore a “housekeeping gene,” while pink boxes denote genes that are less commonly essential in different types of cancer. (C) CRISPR/Cas9-mediated KO of IRF4 or BATF in PEL cell line BC-3 showed that each triggered a strong, but temporally distinct, decrease in live-cell numbers over time, relative to the negative-control sgAAVS1, which is directed to an intergenic safe harbor locus. BC-3/Cas9 cells were transduced with lentiviruses expressing two individual sgRNAs targeting the indicated genes, each delivered at an MOI of 1. See Fig. S2 and S3 for data from three additional PEL cell lines. (D) Western blot analyses of the expression of IRF4, BATF, the IRF4 downstream target MYC, and the loading control GAPDH (glyceraldehyde-3-phosphate dehydrogenase) on day 4 or day 8 following CRISPR/Cas9-mediated KO of IRF4 or BATF as shown in panel C. Results suggest that BATF positively regulates IRF4 expression. See Fig. S1 for quantification across replicates and Fig. S2 and S3 for data from three additional PEL cell lines. (E) Immunoprecipitation of endogenous IRF4 from partially DNase-digested BC-3 nuclear extracts efficiently coprecipitates endogenous KSHV vIRF3, representative of *n* = 3. Quantification of Li-Cor Western results showed that between ∼15% and ∼49% of input vIRF3 coprecipitated with IRF4. See Fig. S4 for reciprocal coimmunoprecipitation of ectopically expressed proteins. HC, heavy chain. (F) CRISPRi-mediated repression of vIRF3 or IRF4 expression triggers a temporally similar decrease in live-cell numbers over time. BC-3/dCas9-KRAB cells were transduced with lentiviruses expressing sgRNAs targeting locations near the TSSs of the indicated genes (“sg-i”), each delivered at an MOI of 1. See Fig. S5 for data from the additional PEL cell line BC-1 and Fig. S6 for rescue experiments that confirmed the specificity of this result. Comparable results were obtained with an additional vIRF3-specific sgRNA (sg-i2; see Fig. 4). (G) Western blot analyses of the expression of vIRF3, IRF4, MYC, and the loading control GAPDH on day 4 of experiments performed as described for panel F. See Fig. S5A for quantification over replicates. We note that knockdown of the targeted genes in these analyses was incomplete because the cells underwent cell death before complete knockdown was observed and samples were collected when a substantial portion of live cells remained. Throughout the figure, error bars represent SEM with numbers of biological replicates indicated. *, *P* < 0.05; **, *P* < 0.01 (paired two-sided Student's *t* tests). n.s., not significant.

Depending on cellular context and its expression level, IRF4 functions as a homodimer or, more often, in complex with other TFs. Since critical binding partners of IRF4 in PEL should be similarly required for the survival of PEL cell lines, we examined the expression and essentiality of established IRF4 co-TFs in our published CRISPR gene essentiality screens (21). Of these, only BATF scored as essential in the majority of PEL cell lines, similarly to IRF4 and MYC (Fig. 1A and B). The CRISPR screens suggested less-significant roles for FLI1 and IKZF1 in smaller subsets of PEL cell lines. Of note, our previous results failed to confirm any dependency of PEL cell lines on IKZF1 (36). BATF is a basic leucine zipper (bZIP) domain containing TF of the AP-1 superfamily. BATF binds to DNA as a heterodimer with JUN family TFs, which can additionally interact with IRF4 or IRF8 (39–41). Our screens did not detect dependencies on a specific JUN family TF, perhaps indicative of a redundancy between expressed family members.

10.1128/mBio.01457-20.1 FIG S1 Quantification of protein expression changes in the Western blots shown in Fig. 1D. Protein expression changes were quantified on day 4 (A) or day 8 (B) of the experiment, using Image Studio software. Expression of the indicated proteins is shown relative to that of GAPDH and matched sgAAVS1 controls. Error bars represent standard errors of the means (SEM) of results from the indicated number of biological replicates. *, *P* < 0.05; **, *P* < 0.01 (*P* values were calculated by paired two-tailed Student’s *t* tests). n.s., not significant. Download FIG S1, TIF file, 3.4 MB. Copyright © 2020 Manzano et al. 2020 Manzano et al. This content is distributed under the terms of the Creative Commons Attribution 4.0 International license.

10.1128/mBio.01457-20.2 FIG S2 BATF is essential in BCBL-1 cells. (A) CRISPR/Cas9-mediated KO of IRF4 or BATF in a BCBL-1 cell clone selected to allow tight and high doxycycline (Dox)-inducible Cas9 expression and transduced with the indicated sgRNAs at MOI 1.5. Dox treatment triggers gene editing, resulting in a strong, and temporally distinct, decrease in live-cell numbers of BCBL-1 cells expressing IRF4 or BATF-directed sgRNAs, relative to the negative-control sgAAVS1. Arrows below the panel indicate that differences remained statistically significant. (B) Representative Western blot analyses of expression of IRF4, BATF, the IRF4 downstream target MYC, KSHV vIRF3, and the loading control GAPDH at 1, 2, 3, or 6 days into Dox treatment in the experiments represented in panel A. In the context of IRF4 KO, the BATF antibody consistently detected a shorter band of unknown nature, marked by a red asterisk. *n* = 4. Western blots are quantified over biological replicates in panels C and D. (C and D) Quantification of protein expression changes over replicates for Western blots as shown in panel B. Protein expression changes were quantified on day 3 (C) or day 6 (D) into the experiment, using Image Studio software. Expression of the indicated proteins is shown relative to that of GAPDH and the sgAAVS1 control. Throughout the figure, error bars represent SEM of results from the indicated number of biological replicates. *, *P* < 0.05; **, *P* < 0.01. *P* values were calculated by paired two-tailed Student’s *t* tests. n.s., not significant. Download FIG S2, TIF file, 10.3 MB. Copyright © 2020 Manzano et al. 2020 Manzano et al. This content is distributed under the terms of the Creative Commons Attribution 4.0 International license.

10.1128/mBio.01457-20.3 FIG S3 BATF is essential in the KSHV/EBV-coinfected PEL cell lines BC-1 and BC-2. (A) Experiments were performed as described for Fig. 1C and D, except that a constitutively Cas9-expressing BC-1 cell pool was used. (B) Representative Western blot analyses of the expression of IRF4, BATF, the IRF4 downstream target MYC, KSHV vIRF3, and the loading control GAPDH on day 3 or day 21 after sgRNA transduction (MOI 1) in the experiments whose results are shown in panel A. In the context of IRF4 KO, the BATF antibody consistently detected a shorter band of unknown nature, marked by a red asterisk. Western blots are quantified over biological replicates in panels C and D. (C and D) Quantification of protein expression changes over replicates for Western blots as shown in panel B. Protein expression changes were quantified on day 3 (C) or day 21 (D) into the experiment, using Image Studio software. Expression of the indicated proteins is shown relative to that of GAPDH and the sgAAVS1 control. (E) Experiments were performed as described for Fig. 1C and D, except that a constitutively Cas9-expressing BC-2 cell pool was used. *n* = 3. (F) Representative Western blot analyses of the expression of IRF4, BATF, the IRF4 downstream target MYC, KSHV vIRF3, and the loading control GAPDH on day 3 or day 5 after sgRNA transduction (MOI 1) in the experiments whose results are shown in panel E. In the context of IRF4 KO, the BATF antibody consistently detected a shorter band of unknown nature, marked by a red asterisk. Western blots are quantified over biological replicates in panels G and H. (G and H) Quantification of protein expression changes over replicates for Western blots as shown in panel F. Protein expression changes were quantified on day 3 (G) or day 5 (H) into the experiment, using Image Studio software. Expression of the indicated proteins is shown relative to that of GAPDH and the sgAAVS1 control. Throughout the figure, error bars represent SEM of results from the indicated number of biological replicates. *, *P* < 0.05; **, *P* < 0.01. *P* values were calculated by paired two-tailed Student’s *t* tests. n.s., not significant. Download FIG S3, TIF file, 16.7 MB. Copyright © 2020 Manzano et al. 2020 Manzano et al. This content is distributed under the terms of the Creative Commons Attribution 4.0 International license.

10.1128/mBio.01457-20.4 FIG S4 vIRF3 associates with IRF4. Ectopically expressed vIRF3 and IRF4 coimmunoprecipitate in 293T. 293T cells were cotransfected with a plasmid expressing FLAG-tagged vIRF3 or an empty vector and yeast chitin-binding domain (CBD)-tagged IRF4 or vitamin K epoxide reductase complex subunit 1 (V1, negative control). Protein complexes were precipitated with anti-FLAG antibody or chitin beads and immunoblotted with anti-FLAG and anti-CBD antibodies. Download FIG S4, TIF file, 3.1 MB. Copyright © 2020 Manzano et al. 2020 Manzano et al. This content is distributed under the terms of the Creative Commons Attribution 4.0 International license.

10.1128/mBio.01457-20.5 FIG S5 KSHV vIRF3 is a candidate for an essential cofactor and regulator of IRF4. (A) Quantification of protein expression changes across replicates of Western blots shown in Fig. 1G. Protein expression of the indicated proteins was quantified using Image Studio software and is shown relative to that of GAPDH and the sgAAVS1 control. (B) Experiments were performed as described for Fig. 1F except that constitutively dCas9-KRAB-expressing BC-1 cells were used. (C) Representative Western blot analyses of the expression of vIRF3, IRF4, MYC, and the loading control GAPDH, on day 3 of experiments were performed as described for panel B. Treatment with TPA was included as a control for the analyses whose results are shown at the bottom of the panel. (D) Quantification of protein expression changes across replicates of Western blots shown in Fig. S5C. Protein expression of the indicated proteins was quantified using Image Studio software and is shown relative to that of GAPDH and the sgAAVS1 control. Throughout the figure, error bars represent SEM of results from the indicated number of biological replicates. *, *P* < 0.05; **, *P* < 0.01. *P* values were calculated by paired two-tailed Student’s *t* tests. n.s., not significant. Download FIG S5, TIF file, 6.1 MB. Copyright © 2020 Manzano et al. 2020 Manzano et al. This content is distributed under the terms of the Creative Commons Attribution 4.0 International license.

10.1128/mBio.01457-20.6 FIG S6 vIRF3 and IRF4 have distinct roles in PEL cells. (A to C) The experiments whose results are shown in Fig. 1F and G were repeated in naïve BC-3 cells, to exclude dCas9-KRAB-independent sgRNA toxicity (A), and in BC-3 dCas9-KRAB cell lines transduced to overexpress vIRF3 with a C-terminal 3XFLAG tag (B) or IRF4 (C). These experiments confirmed the specificity of the CRISPRi approach and showed that vIRF3 and IRF4 serve nonredundant functions. Throughout the figure, error bars, SEM, and *P* values were calculated by paired two-tailed Student’s *t* tests (*, *P* < 0.05; **, *P* < 0.01). n.s., not significant. Download FIG S6, TIF file, 5.3 MB. Copyright © 2020 Manzano et al. 2020 Manzano et al. This content is distributed under the terms of the Creative Commons Attribution 4.0 International license.

To test the dependency of PEL cell lines on BATF, we first inactivated BATF or IRF4 by transducing an EBV-negative PEL cell line that we had previously engineered to express Cas9 (BC-3/Cas9) cells with lentiviruses encoding single guide RNAs (sgRNAs). sgRNAs were delivered at equal multiplicities of infection (MOI). As we have previously demonstrated (21, 36), functional knockout of IRF4 (“IRF4 KO”) led to a dramatic loss of cellular viability, which was accompanied by reduced expression of MYC (Fig. 1C and D; see also Fig. S1 in the supplemental material). Targeting BATF also resulted in a loss of viability; however, this phenotype was significantly delayed compared to the IRF4 KO phenotype (Fig. 1C). Similar results were obtained in three other PEL cell lines, including EBV-negative PEL cell line BCBL-1 (Fig. S2), and two EBV-positive PEL cell lines, i.e., BC-1 and BC-2 (Fig. S3). Our validation of BATF dependency in BC-1 suggests that the results determined for BATF in our BC-1 CRISPR screen represented false negatives (Fig. 1A).

The observed slower loss of viability upon BATF KO in the PEL cell lines was not due to a slower loss of BATF expression upon sgRNA delivery, since BATF protein levels were strongly reduced at the time that sgRNA-dependent inactivation of IRF4 was observed (Fig.1D; see also Fig. S1A, S2B and C, S3B and C, and S3F and G). This delay was also not due to potential functional redundancies with other BATF family members, since BATF2 and BATF3 are not expressed, at least in PEL cell lines BC-3, BCBL-1, and HBL-6 (Fig. 1A [42]). BATF2 and BATF3 are also not upregulated upon BATF KO (see below). Interestingly, the decrease in cellular viability seen upon BATF KO coincided with a clear reduction in IRF4 expression (Fig. 1D; see also Fig. S1B, S2B and D, and S3B, D, F, and H), suggesting that BATF may promote IRF4 expression in PEL cells. The discrepant phenotypes of IRF4 and BATF KO cells suggest that BATF might be an IRF4 cofactor that is required for some but not all oncogenic functions of IRF4 in PEL cells.

### KSHV vIRF3 is in complex with IRF4.

Given the distinct phenotypes of IRF4 and BATF KO and the absence of other cellular candidates for IRF4 interacting partners that scored as essential in the majority of PEL cell lines, we also considered viral latent proteins as possible cofactors of IRF4. Of these, vIRF3 is the most likely candidate, because it exhibits B cell-specific expression and nuclear localization, has partial homology to cellular IRFs, can interact with cellular IRFs, and is essential in PEL cell lines (11, 43–45). We first tested if IRF4 and vIRF3 associate physically with each other. Endogenous IRF4 efficiently coprecipitated nuclear vIRF3 in BC-3 (Fig. 1E), suggesting that a large portion of vIRF3 was in complex with IRF4. Similarly to published results for IRF5 (44), available vIRF3 antibodies were unable to precipitate IRF4, but reciprocal pulldown was obtained using tagged vIRF3 in ectopic expression studies (Fig. S4). Together, these binding studies implicated vIRF3 as a viral co-TF of IRF4 in PEL cells.

### vIRF3 knockdown phenocopies IRF4 inactivation.

If vIRF3 is a key cofactor of IRF4 in PEL, then loss of vIRF3 should affect cellular viability similarly to IRF4 KO. To directly compare the requirements for vIRF3 and IRF4, we sought to suppress each TF using CRISPR interference (CRISPRi [46, 47]). In CRISPRi, endonuclease-dead Cas9 (dCas9) is fused to the transcriptional repressor domain Krüppel-associated box (KRAB) and directed near the transcription start site (TSS) of target genes via sgRNAs, which induces epigenetic silencing of transcription (“knockdown/KD”). We used CRISPRi instead of CRISPR/Cas9, since it is well established that targeting the Cas9 endonuclease to high-copy-number loci (such as the multicopy KSHV episome) results in gene function-independent toxicity, which we have also observed for cellular loci in PEL (21, 48, 49). Side-by-side CRISPRi KD of vIRF3 or IRF4 affected live-cell counts in BC-3/dCas9-KRAB cells to similar degrees at all time points (Fig. 1F and G; see also Fig. S5A). Importantly, CRISPRi-mediated KD of vIRF3 resulted in a significant downregulation of IRF4, which suggests that vIRF3 may promote IRF4 expression. Comparable results were obtained in KSHV/EBV-coinfected PEL cell line BC-1 (Fig. S5B). Inhibition of IRF4 resulted in significantly reduced expression of vIRF3 in some but not all of the settings described above (Fig. 1G; see also Fig. S2B and C, S3B and C, S3F and G, S5A, S5C and D). The specificity of our CRISPRi approach was confirmed by significant rescue of BC-3 cell viability upon reexpression of the targeted TF (Fig. S6). In contrast, overexpression of IRF4 was not able to compensate for the loss of vIRF3 and vice versa. Thus, IRF4 and vIRF3 have nonredundant essential roles in PEL cells and vIRF3 does not simply serve to functionally overexpress an IRF4-like activity.

### IRF4, BATF, and vIRF3 have overlapping genome occupancy.

**FIG 2:**
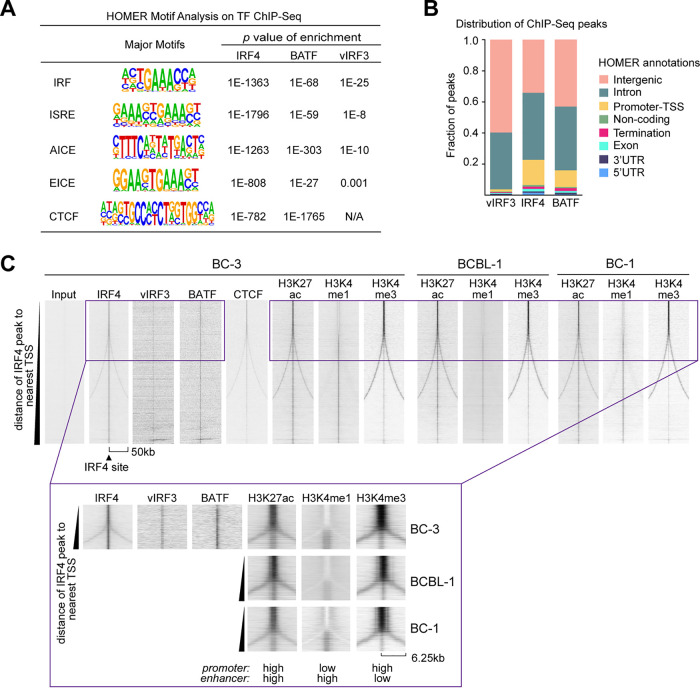
IRF4, vIRF3, and BATF cooccupy genomic sites. (A) HOMER motif analysis identifies significant enrichment of the IRF motif (i.e., the IRF4 binding site) and the ISRE and AICE motifs in BC-3 IRF4, BATF, and vIRF3 ChIP-Seq data. We also detected enrichments for the composite EICE motif and the CTCF binding site. (B) Distribution of location annotations of ChIP-Seq peaks as determined by HOMER. By default, HOMER considers the −1-kb to +100-bp region of the TSS “Promoter-TSS” and considers the −100-bp to +1-kb region of the transcriptional termination site “Termination.” UTR, untranscribed region. (C) IRF4 ChIP-Seq peaks in BC-3 were sorted by the distance (scale = 50 kb on either end) to the nearest TSS and assessed for cooccupancy with vIRF3, BATF, and CTCF as well as nucleosome modification by H3K27ac, H3K4me1, and H3K4me3 in BC-3, BCBL-1, and BC-1 cells. This analysis was performed using EaSeq (80). The insets at the bottom of the panel show enlarged versions of the boxed portion at the top of the heat map, to highlight differential chromatin modifications at promoters and enhancers. Note that the lack of diverging signal (“fork”) in the vIRF3 or BATF ChIP-Seq compared to the IRF4 ChIP-Seq might be due to the inefficient performance of the antibodies in ChIP experiments.

To gain unbiased insight into potential regulatory roles of IRF4, BATF, and vIRF3 in PEL cells, we performed chromatin immunoprecipitation coupled with next-generation sequencing (ChIP-Seq) for each endogenous TF in BC-3 cells. ChIP-Seq experiments yielded reproducible binding site occupancy over replicates in each case (Fig. S7A). We note that available vIRF3 antibodies performed poorly in ChIP-Seq, resulting in relatively suboptimal data sets for vIRF3 that identified an average of only 1,842 peaks, compared to 18,337 for IRF4 and 5,193 for BATF. For highest confidence, we therefore combined available vIRF3 or BATF replicates for subsequent analyses (Table S1 [all supplemental tables are available on Mendeley under https://doi.org/10.17632/9h4xyfnvny.1]). HOMER motif analysis (50) revealed that detected peaks for each TF were strongly enriched for the IRF motif (GAAA, the binding site of all cellular IRFs), the interferon-sensitive response element (ISRE, representing tandem IRF motifs that are occupied by two IRFs), and the AP-1/IRF composite element (AICE) that is generally cooccupied by IRF4 or IRF8 and BATF/JUN (Fig. 2A; see Table S2 on Mendeley for complete results). We additionally detected significant enrichments of the Ets/IRF composite motif (EICE), suggesting that IRF4 may occupy these sites with an Ets family member. *SPI1* (also referred to as PU.1) is the most established Ets family co-TF of IRF4. However, SPI1 is not expressed in PEL cells (Fig. 1A [42, 51, 52]). Another Ets family TF, FLI1, has recently been shown to cooccupy sites with IRF4 in multiple myeloma (31). Since FLI1 is expressed in PEL cell lines and scored as essential in several of our CRISPR screens (Fig. 1A), IRF4 likely occupies the EICE motif together with FLI1 in PEL cell lines. However, fold change values for FLI1-directed sgRNAs in the CRISPR essentiality screens were low (i.e., ∼2-fold to ∼3-fold over 2 weeks [21]), explaining why FLI1 did not meet our stringent statistical cutoff for gene essentiality across PEL cell lines (Fig. 1A and B). The CRISPR data therefore suggest a minor fitness role for FLI1 in PEL cells.

**FIG 3:**
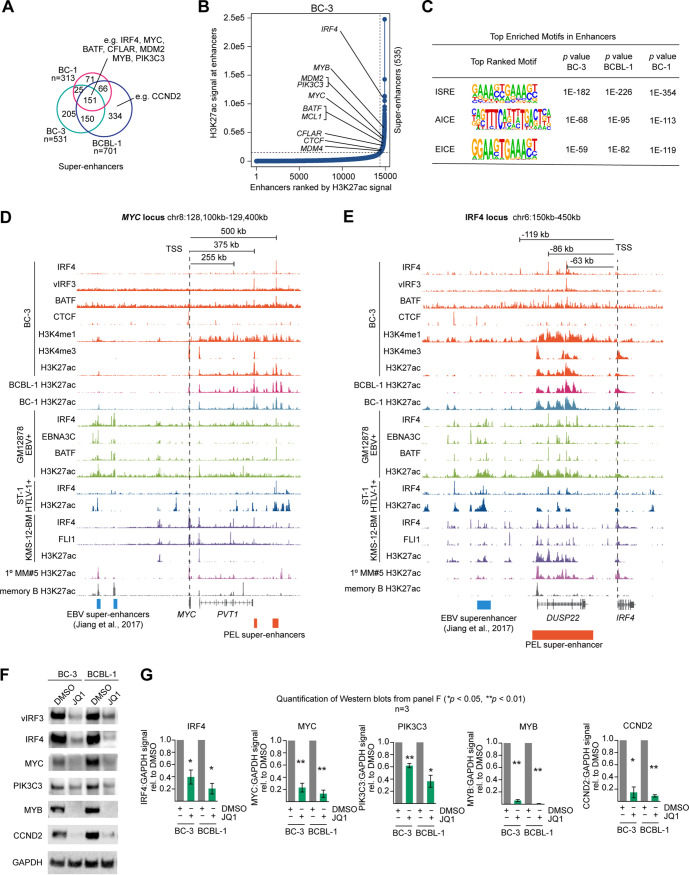
vIRF3, IRF4, and BATF cooccupy super-enhancers (SEs) in PEL cell lines. (A) Overlap of SEs that were identified by ROSE in BC-3, BCBL-1, and BC-1. Candidate SEs for essential genes in PEL are indicated. For complete results in BC-3, BC-1, and BCBL-1, see Table S3. Note that numbers do not add up exactly as described for Table S3, since the HOMER mergePeaks function automatically resolves redundant overlaps by dropping one fragment during analysis; for instance, two distinct shorter SEs from one cell line cannot be logically merged with a longer overlapping SE from a second cell line and plotted in a Venn diagram. (B) Ranking of enhancers and identification of super-enhancers in BC-3, using ROSE. Candidate SEs for essential genes in PEL are indicated. For results in BC-1 and BCBL-1 see Fig. S7B and C. (C) HOMER motif analysis of all enhancers, including both typical enhancers and SEs, determined by ROSE in BC-3, BCBL-1, and BC-1 identified the composite ISRE, AICE, and EICE motifs as the top enriched motifs in each cell line. (D) IRF4, vIRF3, and BATF occupancy and nucleosome modifications by H3K27ac, H3K4me1, and H3K4me3 near MYC in BC-3. Also shown are BCBL-1 and BC-1 H3K27ac marks. For reference, the IRF4, EBNA3C, and H3K27ac ChIP-Seq marks in the LCL GM12878 (from ENCODE) are shown in green; data from the ATLL cell line ST-1 are shown in dark blue; and tracks from the multiple myeloma cell line KMS12-BM, a primary case of multiple myeloma (1° MM#5), and primary memory B cells are shown in shades of purple. The MYC EBV-SEs previously identified in LCL (60) are shown at the bottom of the panel. PEL SEs from BC-3 are indicated by orange boxes. Published data sets (GSE52632, GSE65516, PRJEB25605, and GSE94732) were downloaded from NCBI. (E) Data are presented as described for panel D but with a focus on the region upstream of the IRF4 locus, representing the candidate IRF4 SE in PEL (orange box at bottom). The location of the IRF4 EBV-SE region in LCL is shown at the bottom of the panel (Jiang et al.; 60). (F) Western blot analyses of the expression of essential genes IRF4, vIRF3, MYC, PIK3C3, MYB, and CCND2 2 days after treatment of BC-3 or BCBL-1 cells with 1 μM JQ1. (G) Quantification of Western blots as described for panel F, over n = 3 biological replicates. Error bars represent standard errors of the means (SEM). *, P < 0.05; **, P < 0.01 (paired two-sided Student's t tests).

10.1128/mBio.01457-20.7 FIG S7 vIRF3, IRF4, and BATF cooccupy sites in the genome. (A) Distribution of ChIP-Seq peaks for IRF4, BATF, and vIRF3 for ChIP-Seq replicates. Input controls and individual replicates are compared to peaks from combined analyses of the replicates. (B and C) Ranking of enhancers and identification of super-enhancers in BCBL-1 (B) and BC-1 (C), using ROSE. Candidate SEs for essential genes in PEL are indicated. For results in BC-3, see Fig. 3B. (D and F) Overlap of BC-3 SEs with BC-3 ChiP-Seq peaks for IRF4 from the most sensitive replicate (replicate 3) (D) or from combined replicates for BATF (E) or vIRF3 (F) determined using the HOMER mergePeaks function. Note that the lower quality of our BATF and vIRF3 ChIP-Seq datasets than of the data set corresponding to IRF4 very likely resulted in lower sensitivity and therefore in reduced overlap of SEs. (G and H) IRF4, vIRF3, and BATF occupancy and nucleosome modifications by H3K27ac, H3K4me1, and H3K4me3 near the essential genes *MYB* (G) and *PIK3C3* (H) in BC-3. Also shown are BCBL-1 and BC-1 H3K27ac marks. As references, the IRF4, EBNA3C, and H3K27ac ChIP-Seq marks in the LCL GM12878 (from ENCODE) are shown in green; data from the ATLL cell line ST1 are shown in dark blue; and tracks from the multiple myeloma cell line KMS12-BM, a primary case of multiple myeloma, and primary memory B cells are shown in shades of purple. Published datasets were downloaded from NCBI (GSE52632, GSE65516, PRJEB25605, and GSE94732). Download FIG S7, TIF file, 12.0 MB. Copyright © 2020 Manzano et al. 2020 Manzano et al. This content is distributed under the terms of the Creative Commons Attribution 4.0 International license.

### vIRF3, IRF4, and BATF predominantly bind to active enhancers.

TFs can promote gene expression by binding to promoter regions or distal enhancer elements that are linked to promoter regions through long-range interactions in three-dimensional (3D) space. The large majority of our vIRF3 (98.6%), IRF4 (83.1%), and BATF (89.3%) peaks were outside annotated promoter-transcription start site (TSS) regions and were instead most often found at intergenic and intronic loci (Fig. 2B). To determine whether these peaks map to enhancers, we performed additional ChIP-Seq experiments for analysis of the histone modifications characteristic of active enhancers (53): histone 3 (H3) with acetylated lysine 27 (H3K27ac), a common mark of actively transcribed chromatin, and H3 with monomethylated lysine 4 (H3K4me1), a feature found mostly at poised and active enhancers. To further distinguish enhancers from promoters, we also profiled modification of H3 by trimethylation of lysine 4 (H3K4me3), which is enriched on promoters.

To visually distinguish promoter-proximal sites from distal sites, we sorted all IRF4 ChIP-Seq peaks in BC-3 according to the distance of each from the nearest TSS. Consistent with the HOMER motif analysis, IRF4 peaks showed cooccupancy by vIRF3 and BATF at any location (Fig. 2C). IRF4 peaks prominently colocalized with H3K27ac marks, which indicates they are commonly located within transcriptionally active chromatin. IRF4 peaks near annotated TSSs showed preferential modification by H3K4me3 over H3K4me1, which is consistent with promoter regions (see inset). In contrast, the larger fraction of IRF4 sites was preferentially modified by H3K4me1 over H3K4me3 and found distal from annotated TSSs and thus represented enhancers. The chromatin modifications found at IRF4 peaks in BC-3 were very similar in PEL cell lines BCBL-1 and BC-1, suggesting highly similar enhancer distributions in these cell lines (Fig. 2C). The idea of a role of IRF4, BATF, and vIRF3 in long-range enhancer-mediated gene regulation in PEL cell lines is further supported by the proximity of IRF4 peaks to peaks for CTCF, a known mediator of chromatin looping (Fig. 2C). Accordingly, HOMER motif analysis also showed that IRF4 and BATF peaks were enriched for the CTCF binding site (Fig. 2A).

### IRF4 occupies almost all PEL super-enhancers.

While most BC-3 IRF4 peaks are found on active enhancers, it is unclear whether IRF4, BATF, and vIRF3 occupy the majority of active enhancers and, in particular, so-called super-enhancers (SEs) in PEL cells. SEs are regulatory hubs of densely clustered enhancer elements, with particularly broad and strong H3K27ac modification and high occupancy by TFs. SEs have been shown to drive expression of critical genes that govern lineage decisions or specific disease states. To address this issue, we identified cellular typical enhancers and SEs in PEL cell lines BC-3, BCBL-1, and BC-1 using the algorithm ROSE (rank ordering of super-enhancers) (Fig. 3A and B; see also Fig. S7B and C and Table S3 on Mendeley), which analyzes the density and intensity of active chromatin H3K27ac marks (32, 33). Identified SE regions were highly similar in the three cell lines, with 151 SEs identified in all three cell lines and the majority of SEs in each cell line shared with at least another PEL cell line (∼61% in BC-3, 77% in BC-1, and 52% in BCBL-1). Since SEs have been shown to drive the high expression of oncogenes in other cancers, we specifically searched for SEs in the vicinity of the oncogenes that we had previously identified as essential in PEL (21). This analysis identified high-confidence candidates for SEs that drive expression of *IRF4*, *CCND2*, *MYC*, *MDM2*, and *CFLAR* (see Fig. 3A and B for results from BC-3 and Fig. S7B and C for results from BCBL-1 and BC-1). Previous reports by us and others have already confirmed the importance of these genes for the survival and proliferation of PEL cells (21, 35, 36, 38, 54). SEs were furthermore present in the vicinity of *BATF* and additional genes that scored as essential in our screens, for example, near *MYB* and *PIK3C3*. MYB is a critical TF during hematopoiesis and was found to be a high-confidence PSOD in our screens. It is deregulated in T-cell acute lymphoblastic leukemia (55, 56) and is essential for the survival of acute myeloid leukemia and chronic myeloid leukemia cell lines (57, 58). *PIK3C3* encodes phosphatidylinositol 3-kinase (PI3K) catalytic subunit type 3, an activator of mTORC1 (59), and is the only PI3K catalytic subunit that confidently scores as essential across PEL cell lines (i.e., median adjusted [adj.] *P* = ∼0.068 [21]).

Almost all (i.e., 534/535) SEs in BC-3 overlapped IRF4 peaks, which implicates IRF4 as a major regulator of PEL SEs (Fig. S7D). Many SEs in BC-3 were also occupied by BATF and vIRF3 (Fig. S7E and F), although these overlap analyses potentially underestimate SE occupancy by BATF and vIRF3, due to the lower quality of these ChiP-Seq data sets than the IRF4 data set. The notion that IRF4, vIRF3, and BATF are the major regulators of PEL enhancers is furthermore supported by HOMER motif analysis of all enhancers in BC-3, BCBL-1, and BC-1 cells as defined by ROSE. This analysis, which was conducted in a blind manner with respect to prior knowledge of TF occupancy, identified the ISRE, AICE, and EICE motifs as the top enriched motifs within PEL enhancers (Fig. 3C; see also Table S4 on Mendeley), i.e., the same sites that were the top enriched motifs in the ChIP-Seq peaks for IRF4, vIRF3, and BATF.

In line with these observations, the identified candidate SEs for the essential genes listed above had prominent peaks for IRF4, vIRF3, and/or BATF in BC-3 cells (examples are shown in Fig. 3D and E; see also Fig. S7G and H), suggesting that the role of IRF4 in these SEs involves both vIRF3 and BATF. Interestingly, SE location and IRF4 occupancy in PEL cell lines were at least in some instances distinct from those seen in other IRF4-dependent cell lines, i.e., the LCL GM12878 (60), multiple myeloma cell line KMS-12-BM (31), and HTLV-1 transformed ATLL cell line ST-1 (27). For example, the candidate PEL SEs for *MYC* map to locations ∼375 and 500 kb downstream of *MYC* and carry prominent vIRF3, IRF4, and BATF ChIP-Seq peaks in BC-3 (Fig. 3D). These regions are largely devoid of H3K27ac marks in GM12878 and KMS-12-BM, although some IRF4, BATF, and EBNA3C occupancy was detected at this location in GM12878. In contrast, the same region represents an SE with prominent H3K27ac modification and IRF4/BATF3 cooccupancy in ST-1 cells. Conversely, the known EBV-SEs (ESEs) located ∼550 kb upstream of *MYC* in GM12878 lack IRF4 occupancy and H3K27ac modification in PEL cells (blue boxes; ESEs were previously defined as the subset of SEs in GM12878 that are occupied by all essential EBNA TFs and by nuclear factor kappa B subunits [60]).

The most likely candidate SE for the *IRF4* gene in all three PEL cell lines maps to the *DUSP22* locus, ∼63 kb upstream of the *IRF4* gene, and carries overlapping peaks for vIRF3, IRF4, and BATF in BC-3 (Fig. 3E). This site is a strong candidate for an SE that could mediate the positive regulation of IRF4 by vIRF3 and BATF as suggested by the experiments described above (Fig. 1D and G; see also Fig. S1 to S3 and S5). This SE appears to be shared at least with the LCL GM12878 and KMS-12-BM (Fig. 3E). However, as with MYC, we did not see any IRF4, vIRF3, or BATF occupancy or H3K27ac modification in PEL of the EBV-SE that has been implicated in positive regulation of IRF4 expression in GM12878 (Fig. 3E, blue box [60]). The SE region near *MYB* in PEL cell lines (∼63 kb downstream of the TSS) is not identified as an SE in the other settings and is clearly marked by H3K27ac only in ST-1 (Fig. S7G). Finally, the SE near *PIK3C3* (∼70 kb downstream of the TSS) is also classified as an SE only in PEL but may have typical enhancer function in the other settings, due to evident H3K27ac modification (Fig. S7H). Together, these findings thus point to both differences and similarities between SE location and SE occupancy by IRF4 and its cellular and viral co-TFs in PEL, LCLs, ATLL, and MM. The most likely explanation for these cell type-specific differences is that SE formation and IRF4 occupancy are dependent on cell type-specific cofactors and/or chromatin accessibility.

For experimental confirmation of SE-mediated positive regulation of *IRF4*, *CCND2*, *MYC*, *MYB*, and *PIK3C3* expression, we analyzed the expression levels of these proteins in BC-3 and BCBL-1 cells after treatment with the BET inhibitor JQ1. BET inhibitors block binding of BET proteins to H3K27ac marks, thereby preventing assembly of the Mediator complex and elongation factors, which in turn disrupts SE-mediated gene expression (26, 27, 32). We indeed observed the expected downregulation of each of the tested proteins in JQ1-treated samples (Fig. 3F and G). These results also suggest strongly that BET inhibitors can be used to target an SE-mediated oncogenic transcription program in PEL, as was proposed before (38).

### The super-enhancer in the *DUSP22* locus drives IRF4 expression in PEL.

**FIG 4:**
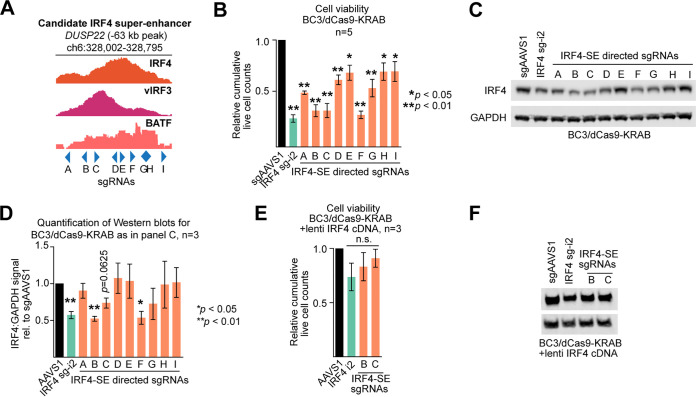
Validation of the IRF4 SE. (A) The region spanning the prominent IRF4 and vIRF3 ChIP-Seq peaks ∼63 kb upstream of the IRF4 TSS was tiled with nine sgRNAs (A to I) for silencing by CRISPRi. (B) CRISPRi-mediated silencing of the candidate IRF4 SE in BC-3. BC-3/dCas9-KRAB cells were transduced with the sgAAVS1 negative-control guide, the IRF4 TSS-directed sgIRF4-i2 positive-control guide, or the 9 individual IRF4-SE-directed sgRNAs shown in panel A, each delivered at MOI 1. Cumulative live-cell counts at day 5 after sgRNA transduction are plotted (*n* = 5). (C) Western analysis of IRF4 expression confirmed significant downregulation of IRF4 for the sgRNAs that also resulted in the most pronounced loss of viability (representative of *n* = 3, see panel D for quantification over replicates). (D) Quantification of Western blots as described for panel C, over *n* = 3 biological replicates. Error bars represent SEM. *, *P* < 0.05; **, *P* < 0.01 (paired two-sided Student's *t* tests). (E) Experiments were performed as described for panel B, except that dCas9-KRAB-expressing BC-3 cells were additionally transduced with a lentiviral expression vector for IRF4. (F) Western blots confirm reexpression of IRF4 in the experiments whose results are shown in panel D (representative result). Throughout the figure, error bars represent SEM with numbers of biological replicates indicated. *, *P* < 0.05; **, *P* < 0.01 (paired two-sided Student's *t* tests). n.s., not significant.

To validate the regulatory importance of the candidate IRF4 SE within the *DUSP22* locus, we targeted an ∼800-bp region centered on the prominent vIRF3/IRF4 peaks ∼63 kb upstream of the IRF4 TSS for epigenetic silencing by CRISPRi (Fig. 4A). Transduction of BC3/dCas9-KRAB cells with 9 individual sgRNAs that tiled this region caused various degrees of toxicity compared to the negative-control sgAAVS1 (Fig. 4B; see Materials and Methods for sgRNA design). The observed phenotype for each sgRNA correlated well with a corresponding reduction of IRF4 expression and was, in some cases, as efficient as that obtained for the positive-control guide IRF4 sg-i2, which is directed near the IRF4 TSS (Fig. 4C and D). The toxicity of the SE-directed sgRNAs was rescued significantly by lentiviral overexpression of IRF4 (Fig. 4E and F), from a cDNA that does not overlap any of the sgRNA binding sites, thereby confirming that the *DUSP22* locus harbors an SE that is essential for PEL cell survival because it drives IRF4 expression in PEL cells. We note that rescue was incomplete at later time points (not shown), which might be explained by incomplete restoration of original IRF4 levels or by control of additional essential genes by this SE.

### IRF4, vIRF3, and BATF are required for IRF4 SE activity.

**FIG 5:**
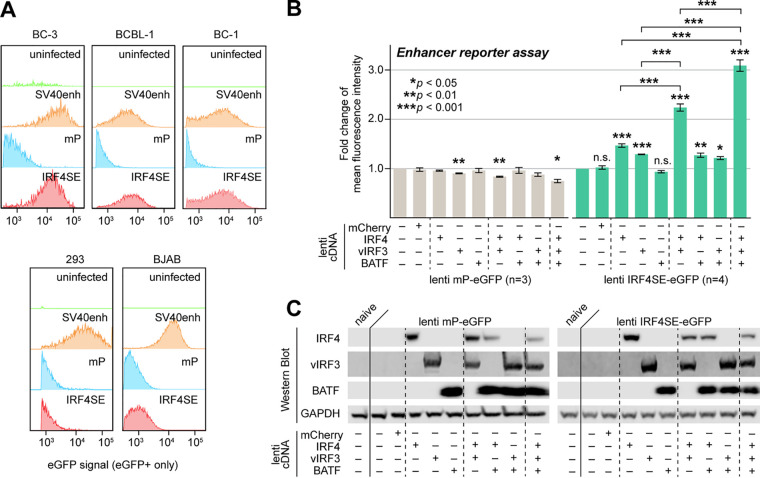
IRF4, vIRF3, and BATF cooperatively promote IRF4-SE activity. (A) A 500-bp sequence centered on the prominent IRF4 and vIRF3 ChIP-Seq peaks ∼63 kb upstream of the IRF4 TSS drove eGFP expression from a lentiviral enhancer reporter (IRF4SE-eGFP) after transduction into PEL cell lines BC-3, BCBL-1, and BC-1 but not the IRF4/vIRF3-negative cell lines 293 and BJAB. Cell lines were transduced at MOI 3 and analyzed 4 days after transduction. Similar reporters containing the SV40 enhancer or a minimal promoter (mP) served as positive or negative controls, respectively. Reporters were titrated by qRT-PCR and, where possible, FACS analysis prior to transduction. Data are representative of results from *n* = 3 biological replicates. (B) The lentiviral IRF4SE or mP eGFP reporters were transduced into 293 cells at MOI 3, together with the indicated combinations of lentiviral expression vectors for vIRF3, IRF4, or BATF. Expression of mCherry served as a negative control. eGFP mean fluorescence intensities were measured by FACS analysis on day 3 after transduction and are shown relative to values from cells that were not transduced with lentiviral cDNA expression vectors. Error bars represent SEM from 3 (mP) or 4 (IRF4SE) biological replicates. *, *P < *0.05; **, *P < *0.01; ***, *P < *0.001 (from paired two-sided Student's *t* tests). n.s., not significant. (C) Western blots from experiments performed as described for panel B, demonstrating ectopic expression of IRF4, vIRF3, and/or BATF as indicated. The blots shown are representative of *n* = 3.

To directly assess the role of IRF4, vIRF3, and BATF in positive regulation via the IRF4-SE, we cloned a 500-bp fragment centered on the IRF4, vIRF3, and BATF ChIP-Seq peaks ∼63 kb upstream of the IRF4 TSS into an insulated lentiviral enhanced green fluorescent protein (eGFP) reporter (pLS-IRF4SE-eGFP [61]). Transduction of the IRF4-SE reporter virus into three different PEL cell lines (BC-3, BCBL-1, and BC-1) resulted in eGFP expression levels comparable to that seen with a positive-control simian virus 40 (SV40) enhancer reporter (Fig. 5A). In contrast, the IRF4-SE reporter was not active after transduction of 293 or BJAB, two cell lines that do not express IRF4 or vIRF3.

We next tested whether the IRF4-SE reporter could be activated by ectopic IRF4, BATF, and/or vIRF3 expression in 293 cells. For this, we cotransduced 293 with a minimal promoter (mP) control reporter or the IRF4-SE reporter lentivirus and various combinations of lentiviruses expressing IRF4, vIRF3, or BATF (Fig. 5B and C). None of the TF combinations induced the minimal promoter reporter. In contrast, IRF4 or vIRF3, but not BATF, reproducibly resulted in modestly increased eGFP expression from the IRF4-SE reporter (∼1.5- and 1.3-fold, respectively; Fig. 5B). Of the pairwise TF combinations, only coexpression of IRF4 and vIRF3 resulted in an additional increase (∼2.2-fold compared to reporter-only controls). Finally, coexpression of all three TFs resulted in the highest level of IRF4-SE reporter expression (∼3.1-fold compared to reporter-only controls). The nonadditive increases in reporter expression strongly point to a cooperative mode of gene activation rather than independent action of these three TFs. These data are consistent with our observation that both vIRF3 and BATF promote the expression of IRF4 in PEL cells. These data furthermore raise the possibility that KSHV uses vIRF3 to initiate an autoregulatory feedback loop that drives overexpression of IRF4 in PEL. Finally, it is likely that similarly cooperative regulation by IRF4, vIRF3, and BATF takes place at other cooccupied SEs.

### IRF4, vIRF3, and BATF promote the expression of many essential genes.

For an independent analysis of the roles of IRF4, vIRF3, and BATF, we performed mRNA sequencing (mRNA-Seq) upon inactivation of these TFs in PEL cell line BC-3 (see Table S5 on Mendeley). We used CRISPRi to repress vIRF3 (Fig. S8A) and CRISPR/Cas9 to inactivate IRF4 or BATF (Fig. S8B), which ensured the most efficient loss of function in each case. We transduced lentiviral sgRNAs vectors at MOIs of 3, leading to transduction of the vast majority of cells. We assessed the impact of BATF KO on mRNA expression both early (day 3), at a time point matched with sgIRF4, and later (day 8), when sgBATF began to affect cellular viability. Induction of cell death by diverse stimuli has been reported to induce global mRNA decay (62, 63). Therefore, to control for effects due to loss of cellular viability rather than due to specific perturbation, we additionally performed mRNA-Seq after CRISPR/Cas9-induced MDM4 KO, in parallel with IRF4 and BATF KO (Fig. S8B). Our gene essentiality screens have identified MDM4 as essential in PEL cells, but the mechanism underlying MDM4 essentiality is expected to be unrelated to IRF4, BATF, or vIRF3 (21). Principal-component analysis (PCA) of all mRNA-Seq data from BC-3/Cas9 clearly showed that the MDM4 KO expression profiles at day 16 (∼50% viability) were distinct from those of IRF4 or BATF KO cells (Fig. S8C). We furthermore note that an additional unrelated lethal genetic perturbation in BC-3, which triggers a rapid loss of viability similar to that triggered by sgIRF4, also had a strikingly distinct effect on gene expression (M. Manzano and Eva Gottwein, unpublished data). These observations together suggest that the gene expression changes that we detected following IRF4, vIRF3, and BATF inactivation are substantially specific to these genetic perturbations rather than due to impaired viability.

10.1128/mBio.01457-20.8 FIG S8 vIRF3, IRF4, and BATF induced gene expression changes. (A) Controls run in parallel with the vIRF3-mRNA-Seq experiment show cumulative cell viability (left) and vIRF3 expression in Western blots (right) for BC-3 dCas9-KRAB cells upon transduction with lentiviruses expressing sgRNAs for AAVS1 or vIRF3 (sg-i7 or sg-i8) at MOI 3. Samples for mRNA-Seq were taken on day 4 after transduction. *n* = 3 technical replicates were included in this experiment; for biological replicates, see Fig. 1. Error bars represent SEM. (B) Controls run in parallel with the IRF4, BATF, and MDM4-mRNA-Seq experiments showed cell viability (left) and IRF4, BATF, and MDM4 expression in Western blots (right) for BC-3 Cas9 cells upon transduction with lentiviruses expressing sgRNAs for AAVS1, IRF4, BATF, or MDM4 at MOI 3. Samples for mRNA-Seq were taken 3 (all sgRNAs), 8 (sgBATF, sgMDM4, and sgAAVS1 only), and 16 days (sgMDM4 and sgAAVS1 only) after sgRNA transduction. *n* = 3 technical replicates were included in this experiment; for biological replicates, see Fig. 1. Error bars represent SEM. (C) Principal-component analysis (PCA) of the CRISPR/Cas9 mRNA-Seq datasets for KO of IRF4, BATF, and MDM4. (D) MA plots of gene expression changes in BC-3 cells transduced with vIRF3 sg-i8, IRF4 sg1, BATF sg2 (day 3 and day 8), or sgMDM4 (day 16) compared to their matched sgAAVS1 controls. Genes that are differentially expressed (adj. *P < *0.05, no fold change cutoff) are indicated in pink; all KSHV genes are indicated in blue. The extent of KSHV reactivation in BC-3 is addressed below. (E) Principal-component analysis (PCA) of the CRISPR/dCas9-KRAB mRNA-Seq datasets for KD of vIRF3 using sg-i7 or sg-i8. Note that almost all variance is observed in principal component 1 (PC1, 97%). (F) Transcriptome-wide comparison of mRNA fold changes induced by the two vIRF3-targeting sgRNAs sg-i7 and sg-i8. Download FIG S8, TIF file, 14.7 MB. Copyright © 2020 Manzano et al. 2020 Manzano et al. This content is distributed under the terms of the Creative Commons Attribution 4.0 International license.

**FIG 6:**
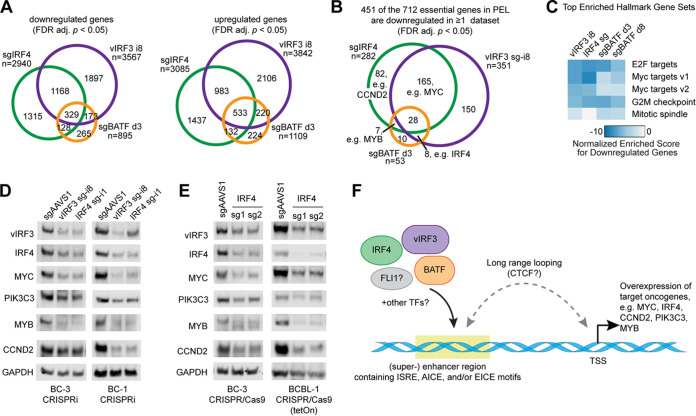
Inactivations of IRF4, vIRF3, and BATF have similar consequences for gene expression. (A) Overlap of differentially expressed genes (FDR-adj. *P < *0.05, no fold change cutoff) in mRNA-Seq experiments after IRF4 KO, BATF KO, or vIRF3 KD in BC-3 cells, compared to matched sgAAVS1 controls. d3, day 3. (B) Downregulation (FDR-adj. *P < *0.05, no fold change cutoff) of a large subset of the 712 genes that we had previously identified as essential across PEL cell lines (21) in the mRNA-Seq experiments after IRF4 KO, BATF KO, or vIRF3 KD in BC-3 cells, compared to matched sgAAVS1 controls. (C) Heat map showing enrichment of Hallmark gene sets among the downregulated genes, identified using gene set enrichment analysis. The top four enriched categories from any of the data sets are shown across all data sets. (D and E) Western blot analyses of the expression of candidates for vIRF3/IRF4/SE-controlled essential genes (IRF4, MYC, PIK3C3, MYB, CCND2) and loading control GAPDH, following CRISPRi-mediated KD of vIRF3 and IRF4 in BC-3 or BC-1 cells (D) or CRISPR/Cas9-mediated KO of IRF4 in BC-3 or BCBL-1 cells (E), as indicated. Western blots for *n* ≥ 3 biological replicates were quantified, and results are shown in Fig. S9. (F) Proposed working model of the cooperative functions of vIRF3, IRF4, and BATF in the positive regulation of cellular super-enhancers in PEL cell lines.

We observed similar numbers of significantly down- or upregulated genes after inactivation of IRF4, BATF, or vIRF3 (Fig. 6A; see also Fig. S8D). The overexpression of many mRNAs under each set of conditions further supports our interpretation that gene expression changes do not simply reflect global mRNA decay. The results obtained for two independent vIRF3 sgRNAs were highly concordant (Pearson correlation coefficient *R* = 0.95), demonstrating the high specificity of CRISPRi (Fig. S8E and F). For simplicity, data from the more efficient i8 sgRNA, which was also used in the experiment described above, are used here to represent vIRF3 KD. Approximately half of the mRNAs that were down- or upregulated upon IRF4 KO were also significantly and similarly regulated upon vIRF3 KD (Fig. 6A). BATF KO resulted in more subtle gene expression changes on day 3 in terms of magnitude (Fig. S8D). However, the large majority of the differentially downregulated (i.e., 70%) or upregulated (i.e., 79%) genes in BATF KO cells were also significantly and similarly affected by KO of IRF4 and/or KD of vIRF3.

10.1128/mBio.01457-20.9 FIG S9 Quantification of protein expression changes in Western blots shown in Fig. 6. (A to D) Protein expression changes seen in the experiments represented in Fig. 6D (A and B) and Fig. 6E (C and D) were quantified over biological replicates, using Image Studio software. Expression of the indicated proteins is shown relative to that of GAPDH and the sgAAVS1 control. Note that these Western blots were run on lysates that are shared with analyses shown in other panels, as indicated in the figure. Error bars represent SEM of results from the indicated number of biological replicates. *, *P* < 0.05; **, *P* < 0.01. *P* values were calculated by paired two-tailed Student’s *t* tests. Download FIG S9, TIF file, 6.9 MB. Copyright © 2020 Manzano et al. 2020 Manzano et al. This content is distributed under the terms of the Creative Commons Attribution 4.0 International license.

On the basis of our hypothesis that IRF4, vIRF3, and BATF function on SEs to drive the expression of essential oncogenes in PEL cells, we investigated the regulation of the 712 genes we had previously designated essential in PEL cells in our mRNA-Seq data sets. Indeed, 451, i.e., ∼63% of the 712 essential genes, were downregulated under at least one set of conditions. MYC was downregulated following inactivation of either IRF4 or vIRF3. Indeed, gene set enrichment analysis (GSEA [64]) revealed highly significant enrichments of “MYC targets” and other categories broadly associated with cellular proliferation and viability among the downregulated genes (Fig. 6C; see also Table S6 on Mendeley). Approximately 20% of the 451 downregulated essential genes in Fig. 6B are known targets of MYC that contribute to the MYC-related GSEA signatures. This finding suggests that the downregulated essential genes are likely not all directly regulated by IRF4, vIRF3, and/or BATF but also include downstream effectors of MYC. Nevertheless, downregulated genes are likely to include several with SEs under direct control by IRF4, BATF, and/or vIRF3. For example, our mRNA-Seq data confirm downregulation of CCND2 and MYB mRNAs, in at least a subset of the conditions (see Table S6 on Mendeley). Upon inactivation of vIRF3 and BATF, we furthermore observed significant downregulation of IRF4 mRNA expression, consistent with positive regulation of IRF4 expression by vIRF3 and BATF. In sum, the highly concordant gene regulation patterns of IRF4 and vIRF3 are consistent with the idea that vIRF3 and IRF4 are the major TFs that cooperate to promote PEL survival by driving high expression of many survival genes, including but not limited to *MYC*.

To validate the IRF4/vIRF3-dependent expression of several candidates for downstream effectors that also have prominently IRF4/vIRF3-occupied SEs in their vicinities (Fig. 3B and D to E; see also Fig. S7B, C, G, and H), we performed quantitative Western blot analyses of MYC, CCND2, MYB, and PIK3C3 after inhibition of vIRF3 or IRF4. For this experiment, we inhibited vIRF3 or IRF4 expression using CRISPRi in PEL cell lines BC-3 and BC-1. We additionally inactivated IRF4 in BC-3 and the additional PEL cell line BCBL-1 using CRISPR/Cas9. In all of these settings, inactivation of vIRF3 or IRF4 led to a significant decrease in the expression of each protein (Fig. 6D and E; see also Fig. S9). Future experiments should validate the SEs that control these genes, similarly to our validation experiments for the IRF4 SE described above.

### vIRF3 and IRF4 might have additional repressive functions.

In addition to their roles in SE-mediated activation of PEL oncogenes, vIRF3, IRF4, and, at least in some cases, BATF may also function to repress genes, including those whose expression is incompatible with survival and proliferation. Specifically, the mRNA-Seq data set revealed induction of large numbers of interferon-stimulated genes (ISGs) upon vIRF3 KD and IRF4 KO, which contributes to highly significant enrichment observed in gene sets related to interferon alpha (IFN-α) and IFN-γ responses (Fig. S10A and B; see also Table S6 on Mendeley). ISG induction following vIRF3 KD in PEL cells was reported previously (44). Given that IFN treatment is toxic to PEL cells (35), the observed ISG induction might contribute to loss of cell viability. However, we note that this induction of ISGs is almost certainly independent of type I IFN production since PEL cell lines lack any baseline IFN expression (35) and type I IFN expression is also not seen upon TF inactivation (see Table S5 on Mendeley). In contrast, our data confirm the reported induction of IFN-γ mRNA upon inactivation of vIRF3 and show a more modest induction of IFN-γ upon inactivation of IRF4 (see Table S5 on Mendeley) (65). The observed IFN-γ response signature represented in Fig. S10A and B was furthermore found to be related to an induction of downstream targets of IFN-γ, i.e., HLA genes and CIITA (Fig. S10C), consistent with a previous report on vIRF3 (65). Inactivation of vIRF3, IRF4, or BATF also caused the induction of several proapoptotic genes and the cell cycle inhibitor CDKN1A/p21 and affected the expression of transcription factors involved in B cell differentiation (Fig. S10C; see also Table S5). These potentially repressive functions of IRF4 and/or vIRF3 should be explored further, in terms of their functional roles and mechanisms of regulation.

10.1128/mBio.01457-20.10 FIG S10 IRF4 and vIRF3 may have functions in addition to SE regulation. (A) Heat map showing enrichment of Hallmark gene sets among the upregulated genes, identified using gene set enrichment analysis (GSEA). The top four enriched categories from any of the datasets are shown across all datasets. (B) Relative gene expression (z score) levels of all genes in the GSEA Hallmark gene sets “interferon alpha response” and “interferon gamma response” across the mRNA-Seq datasets. (C) Relative gene expression (z score) levels of all major histocompatibility complex genes (top) and lineage-defining transcription factors (PRDM1, XBP1, and BCL6) (bottom) involved in B cell differentiation across the mRNA-Seq datasets. (D) Immunofluorescence staining of K-bZIP (red) or nuclei (DAPI, blue) of BC-3 dCas9-KRAB cells transduced with sgAAVS1, vIRF3 sg-i8, or IRF4 sg-i1 sgRNAs (day 3 after transduction). As a positive control for KSHV reactivation, lysates from cells treated with 20 ng/ml TPA for 2 days were used. For quantification, see text and Materials and Methods. (E) Western blots for the viral lytic gene K-bZIP in BC-3 and BC-1 dCas9-KRAB cells, 3 days (BC-1) or 4 days (BC-3) after transduction with sgAAVS1, vIRF3 sg-i8, or IRF4 sg-i1. As a positive control for KSHV reactivation, lysates from cells treated with 20 ng/ml TPA for 2 days were used. (F) Day 3 lysates from the experiment represented in Fig. S2B were also probed for K-bZIP and showed detectable KSHV reactivation after Dox-induced CRISPR/Cas9 KO of either IRF4 or BATF in BCBL-1 cells. Download FIG S10, TIF file, 16.6 MB. Copyright © 2020 Manzano et al. 2020 Manzano et al. This content is distributed under the terms of the Creative Commons Attribution 4.0 International license.

Finally, the mRNA-Seq data suggested nearly genome-wide induction of lytic KSHV transcripts upon TF inactivation (Fig. S8D). Importantly, however, this signature stemmed from only a minor percentage of lytic cells, which confounds the bulk sequencing data, since induction of the early viral lytic gene K-bZIP was seen in only <1% of the BC-3 culture by immunofluorescence analysis (Fig. S10D) and was not detected by bulk Western blotting upon IRF4 or vIRF3 KD in either BC-3 or BC-1 cells (Fig. S10E). Thus, at least in BC-3 and BC-1 cells, lytic reactivation did not substantially contribute to the loss of cellular viability in the bulk of the culture upon suppression of vIRF3 or IRF4. We note that, in contrast to BC-3 and BC-1 cells, inactivation of IRF4 or BATF in BCBL-1 resulted in a more robust reactivation phenotype (Fig. S10F), suggesting that IRF4 and MYC could in fact govern the latent/lytic switch in this cell line, reminiscent of the recently reported role of MYC in the maintenance of EBV latency and consistent with studies of IRF4 and MYC in KSHV infection (54, 66, 67).

Taken together, our data show that vIRF3, IRF4, and BATF are key oncogenic regulators of SE-mediated gene expression in PEL and might have additional roles in the repression of several likely toxic genes, such as ISGs, and in the maintenance of KSHV latency.

## DISCUSSION

IRF4 has recently emerged as a major player in KSHV-induced B cell transformation. Here, we have begun to characterize the regulation of IRF4 expression and the essential functions of IRF4 in PEL cell survival and proliferation. Our phenotypic, interaction-based, ChIP, and gene expression studies all support the model that IRF4 exerts key regulatory functions for enhancers and SEs, where it cooperates with cellular BATF and viral vIRF3 to drive expression of many essential genes (Fig. 6F).

With almost all PEL SE regions occupied by IRF4 and IRF4-binding sites identified as the top enriched motifs in PEL enhancers, our data suggest that IRF4 is the dominant regulator of SE-mediated gene expression in PEL cell lines. Consistent with the role of SEs in activation of cell/disease identity genes in other settings, the PEL SEs include IRF4-, BATF-, and vIRF3-bound regions in close proximity of essential genes, including *IRF4* itself, *CCND2*, *MYC*, *MYB*, and *PIK3C3*. Our findings likely explain the reported high sensitivity of PEL cell lines to BET bromodomain inhibitors such as JQ1, which release BET proteins BRD2 and BRD4 from enhancer elements (35, 38).

While we detected IRF4-BATF composite sites in our ChIP-Seq data, our loss of function and gene expression data suggest that the role of BATF in PEL cells is perhaps less substantial than the role of IRF4 and vIRF3. Importantly, BATF KO cells continue to survive until IRF4 levels decrease. Thus, one essential function of BATF is to positively regulate IRF4, most likely through the validated IRF4-SE located in the *DUSP22* gene. To our knowledge, this is the first direct demonstration that BATF can regulate IRF4 expression in any setting. We speculate that BATF may similarly promote IRF4 overexpression in LCLs, where BATF occupies both an upstream EBV-SE and the IRF4-SE identified here in PEL cells. Importantly, the PEL-IRF4-SE has not yet been investigated for its importance for IRF4 expression and essentiality in LCLs.

Our data in PEL suggest that positive regulation of IRF4 expression by vIRF3 and BATF occurs at the level of transcription, since the IRF4 mRNA was downregulated upon KD of vIRF3 or KO of BATF in BC-3 cells. Furthermore, these TFs cooperatively drive expression of an IRF4-SE reporter without any effects on their mutual expression levels. We note, however, that we cannot rule out the possibility that these proteins enhance each other’s stability in BC-3 cells through complex formation. Future study of the molecular mechanism of the effect of vIRF3 on IRF4-dependent transcriptional regulation might further clarify the roles of these TFs. Importantly, vIRF3 lacks nearly all of the conserved amino acids that mediate recognition of the IRF motif 5′-GAAA by cellular IRFs, making an IRF-like interaction of vIRF3 with the IRF motif highly unlikely. A possible scenario is that vIRF3 is recruited to DNA via interaction with IRF4 and/or BATF, similarly to EBNA3C (68).

Interestingly, motif analysis of enhancer H3K27ac by ChIP-Seq implicated the Ets family TF FLI1 as an additional candidate for a regulator of cellular enhancers in PEL, reminiscent of the recently reported cooperation of FLI1 with IRF4 on SEs in multiple myeloma (31). On the basis of our published CRISPR screens, the contribution of FLI1 is most likely subtle. Perhaps FLI1 and BATF are each important for only a subset of the regulatory roles of vIRF3 and IRF4 and therefore their inactivation results in a less dramatic phenotype than inactivation of IRF4 or vIRF3. Future studies should clarify the importance of FLI1 in PEL cells. It would furthermore be interesting to investigate the expression, essentiality, and function of FLI1 in LCL.

The intimate collaboration and comparable levels of importance of IRF4 and vIRF3 suggest that this interaction is key to B cell infection by KSHV. Interestingly, vIRF3 also positively regulates IRF4 expression, most likely through the prominent vIRF3 site on the validated IRF4-SE. With our identification of vIRF3 as an IRF4 co-TF, it is apparent that each of the three human viruses linked to hematologic malignancy (EBV, KSHV, and HTLV-1) encodes at least one protein that usurps IRF4 and/or BATF family members to promote the survival and proliferation of infected cells. The relationships among the genome occupancy, gene expression effects, and phenotypes of vIRF3, IRF4, and BATF are surprisingly reminiscent of the interplay among the EBV EBNA3A/C proteins, IRF4, and BATF in the oncogenic transcriptional reprogramming that occurs in the context of type III EBV latency, observed, for example, in the LCL model (69). Common between these two viral strategies is that both vIRF3 and EBNA3A/C use IRF4 to promote the expression of cellular survival genes such as *MYC* and *IRF4* itself. At least in PEL, positive regulation of IRF4 also requires BATF. The positive regulatory role of oncoviral TFs at the SEs of essential genes may at least be partially shared with the oncogenic HBZ transcription factor encoded by HTLV-1, which has recently been shown to promote SE-dependent BATF3 expression in ATLL (27). While all three models depend on IRF4-mediated enhancer function for survival gene expression, the exact SEs and/or typical enhancers utilized in each setting can coincide or differ at important sites, such as the *IRF4* or *MYC* SEs, respectively. Although most PEL cell lines are coinfected with EBV, these coinfected PEL cells do not express EBNA3/C but instead depend on vIRF3, similarly to EBV-negative PEL cell lines. Therefore, we expect that enhancer regulation by IRF4 in KSHV/EBV-coinfected PEL tumors would be distinct from that seen with LCLs. In support of this, SEs in EBV-positive PEL cell line BC-1 were more similar to those in BC-3 (55% overlap) and BCBL-1 (72% overlap) than to those in LCL (only 26% overlap). This conclusion does not exclude a role for other EBV proteins and/or noncoding RNAs in the pathogenesis of coinfected PEL tumors. Differences in IRF4 occupancy and SE location between the various IRF4-dependent model systems most likely reflect the differential interplay with additional TFs, such as other oncogenic EBNA proteins in the case of LCLs. Candidates for additional regulators of vIRF3/IRF4-dependent transcription in PEL are, in addition to FLI1, the viral LANA and vIRF1 proteins, as well as the vIRF3/vIRF1 interaction partner USP7, all of which have been reported to be essential in PEL cells (12, 70).

Our finding that KSHV-transformed PEL cells use vIRF3 and IRF4 to drive the expression of key oncogenes through SEs raises several fundamental issues. First, it is unclear how SE-dependent chromatin architecture is formed during the process of B cell transformation by KSHV. It is possible that the mere binding of vIRF3 to IRF4 is sufficient to alter site selection on preexisting open chromatin, for example, during B cell differentiation. It is equally probable that KSHV induces a pioneer factor that remodels the host DNA—perhaps vIRF3 itself or even a lytic or cellular protein that is required only for establishment and not for maintenance of chromatin structures during the initial stages of B cell transformation. However, studying these processes will remain a challenge until a physiologically relevant model for B cell transformation by KSHV is established. Second, our genomics data indicate that IRF4, BATF, and vIRF3 likely each have functions that go beyond enhancer regulation. It will be interesting to study the identity of the complexes that each of these TFs participates in as well as the molecular determinants that dictate the positive or, possibly, negative regulatory outcome resulting from the presence of these complexes at specific distal elements or promoter sites. Here, too, differential regulatory outcomes are most likely determined by the context of additional transcriptional cofactors.

Importantly, our results regarding the mechanisms underlying IRF4 dependency could be exploited to devise better treatment strategies for PEL. PEL cell lines are sensitive to immunomodulatory drugs (IMiDs), which are known to antagonize IRF4 expression in PEL cells (35, 36), and to BET inhibitors (38), which function by inhibiting SE-mediated gene expression. IMiDs or BET inhibitors could furthermore be combined with drugs that exploit the dependency of PEL cells on essential IRF4 effectors, such as CDK4/6 inhibitors that target CCND2 dependency and that are highly toxic in PEL cells (21).

In sum, this report identifies cellular IRF4 and BATF and KSHV vIRF3 as the key TFs involved in the SE-mediated activation of essential genes and PSODs in KSHV-transformed PEL cell lines.

## MATERIALS AND METHODS

### Cell lines.

Tumor-derived PEL cell lines were obtained from original sources, where possible, and have been validated by short tandem repeat (STR) profiling and/or PCR detection of the KSHV or EBV genomes (21). PEL cell lines were grown in RPMI 1640 (Corning, Corning, NY) containing 10 μg/ml gentamicin (Life Technologies, Carlsbad, CA), 0.05 mM β-mercaptoethanol (Sigma-Aldrich, St. Louis, MO), and 10% (BC-1 and BCBL-1) or 20% (BC-2 and BC-3) Serum Plus II medium supplement (Sigma, catalog no. 14009C). All PEL cell lines were maintained at concentrations of between ∼2 × 10^5^ and ∼1 × 10^6^ cells/ml. HEK-293T cells (ATCC, Manassas, VA) and HEK-293 cells (from ATCC via the Duke University Medical Center Cell Culture Facility) were cultured in Dulbecco’s modified Eagle medium (Corning) containing 10% Serum Plus II medium supplement and 10 μg/ml gentamicin. All cell lines were grown at 37°C with 5% CO_2_.

### Cloning.

All primers and DNA fragments used for cloning were synthesized by Integrated DNA Technologies (Coralville, IA), and the sequences can be found in Table S7 on Mendeley at https://doi.org/10.17632/9h4xyfnvny.1.

sgRNA constructs used with constitutively expressed Cas9 or dCas9-KRAB were cloned as annealed oligonucleotides into plentiGuide-Puro (a gift from Feng Zhang; Addgene catalog no. 52963), using the BsmBI site as described previously (21). For the design of IRF4-SE-targeting sgRNAs, we focused on the major shared peak for IRF4/vIRF3/BATF in the candidate IRF4-SE, ∼63 kbp upstream of the IRF4 TSS. We selected an ∼800-bp window centered on this peak and used a published sgRNA design tool to predict candidate sgRNA target sites within this sequence (71). High-scoring sites throughout this sequence were chosen, while candidate sites that were within an ∼50-bp window of a higher scoring site were ignored.

sgRNA constructs used with doxycycline (Dox)-inducible Cas9 were cloned into pLX-sgRNA (a gift from Eric Lander and David Sabatini; Addgene catalog no. 50662). To clone other sgRNAs into pLX, we used pLX-sgRNA as a template for overlap PCR and then replaced the sgAAVS1 sequence in the parental vector with the resulting PCR products. For this, PCR 1 fragments were generated using the outer forward primer 2430 and sgRNA-specific reverse primers listed in Table S7. PCR 2 fragments used the outer reverse primer 2431 and sgRNA-specific forward primers listed in Table S7. PCR 1 and 2 were used together as templates in a final PCR step with outer primers 2430 and 2431. Resulting sgRNA-containing PCR fragments were used to replace the corresponding XhoI-NheI fragment of pLX-sgRNA.

IRF4 and vIRF3-3XFLAG cDNA constructs used for rescue of CRISPRi-mediated inhibition of endogenously expressed counterparts (see Fig. S6 in the supplemental material) were cloned into the lentiviral backbone pLC/ZsGreen-P2A-Hygro (72), by replacing ZsGreen. The IRF4 cDNA was amplified from pCW-IRF4-P2A-puro (36) using primers 4264 and 4257. The vIRF3 cDNA was amplified from a cDNA template that was originally amplified from BC-1 genomic DNA and initially cloned into pMSCV-hyg (Clontech), using primers 4203 and 4204. The 3× FLAG tag was amplified from pTRIPZ/KapA-3XFLAG (73) using primers 4205 and 4141. The IRF4 cDNA or the vIRF3 cDNA and the 3XFLAG fragment were subjected to Gibson assembly with the vector fragment that resulted from digestion of pLC/ZsGreen-P2A-Hygro with AgeI and BamHI.

The IRF4, vIRF3, and BATF expression vectors used for enhancer reporter assays (i.e., pLC-IRF4-IRES-Puro, pLC-vIRF3-IRES-BLAST, and pLC-BATF-IRES-Zeo) were based on other newly constructed lentiviral vectors containing various resistance cassettes downstream of an internal ribosome entry site (IRES) element. To initially clone pLC-ZsGreen-IRES-Hyg and pLC-IRF4-IRES-Hyg, we amplified PCR products containing ZsGreen (primers 4087/4308) or the sgRNA-resistant IRF4 expression cassette in pLC-IRF4-P2A-Hyg (from the experiment described above; primers 4087/4309) and an IRES-hygromycin resistance (hygromycin^r^) (primers 4306/4307) fragment. The resulting ZsGreen or IRF4 expression cassettes were then subjected to Gibson assembly with the IRES-hygromycin^r^ cassette and the AgeI/EcoRI fragment of the lentiviral backbone pLC/ZsGreen-P2A-Hygro (72). To construct pLC-vIRF3-IRES-Hyg, an untagged vIRF3 expression cassette was amplified from pLC-vIRF3-3XFLAG-P2A-Hyg (from the experiment described above, primers 4370/4373), digested using AgeI and BamHI, and used to replace the corresponding ZsGreen cassette in pLC-ZsGreen-IRES-Hyg using T4 DNA ligase. Finally, we exchanged the “IRES-hygromycin^r^” cassette of pLC-ZsGreen-IRES-Hyg, using Gibson assembly of a BamHI/EcoRI-digested pLC-ZsGreen-IRES-Hyg vector fragment, with PCR products containing an IRES-puromycin^r^ fragment (amplified using primers 4566 and 4569), an IRES-blasticidin^r^ fragment (amplified using primers 4566 and 3911), or separate PCR products containing IRES and zeocin^r^ fragments (amplified using primers 4566/4567 and 4568/3890, respectively). The resulting vectors were named pLC-ZsGreen-IRES-Puro, pLC-ZsGreen-IRES-BLAST, and pLC-ZsGreen-IRES-Zeo. To clone pLC-IRF4-IRES-Puro, pLC-ZsGreen-IRES-Puro was digested using AgeI and BamHI and ligated with the IRF4-containing AgeI-BamHI fragment from pLC-IRF4-IRES-Hyg. To clone pLC-vIRF3-IRES-BLAST, pLC-ZsGreen-IRES-BLAST was digested using AgeI and BamHI and ligated with the vIRF3-containing AgeI-BamHI fragment from pLC-vIRF3-IRES-Hyg. To clone pLC-BATF-IRES-Zeo, pLC-ZsGreen-IRES-Zeo was digested with AgeI and BamHI and used for Gibson assembly with a PCR product encoding BATF, which was in turn amplified from a gBlock containing the BATF coding sequence (see Table S7), using primers 4087 and 4570.

Lentiviral plasmid vector pCEBZ-VKORC1v1-CBD, expressing chitin-binding domain (CBD)-tagged VKORC1 (variant-1), was described previously (74). An equivalent lentiviral vector expressing IRF4-CBD was generated by PCR amplification of IRF4 coding sequences from cDNA with primers containing NotI and BamHI restriction sites and cloning of the product between same sites of the lentiviral expression vector pCEBZ-RFP-CBD (74) to replace the RFP open reading frame (ORF) and generate pCEBZ-IRF4-CBD.

The lentiviral enhancer construct pLS-mP expressing eGFP under the control of a minimal promoter (61) was pLS-mP (a gift from Nadav Ahituv; Addgene plasmid catalog no. 81225). An mCherry version of pLS-mP (pLS-mP-mCherry) was made by Gibson assembly of a PCR product containing mCherry (amplified using primers 4546 and 4547) with the AgeI/EcoRI vector fragment of pLS-mP. The SV40 enhancer was amplified from pCMVR8.74 using primers 4538 and 4539 and Gibson cloned into XbaI-linearized pLS-mP-eGFP and pLS-mP-mCherry to make pLS-SV40-eGFP and pLS-SV40-mCherry, respectively. Finally, a 500-bp fragment centered on the −63 kb IRF4 SE was amplified directly from BC-3 genomic DNA using primers 4597 and 4598 and subjected to Gibson assembly into XbaI-digested pLS-mP, resulting in pLS-IRF4-SE-eGFP.

### Lentivirus production.

Lentiviruses were packaged using second-generation vectors in HEK293T. For a 150-mm-diameter dish containing ∼70%-confluent cell monolayers, 11 μg of the lentiviral backbone was mixed with 5.48 μg of pMD2.G, 8.24 μg psPAX2, 1 ml Opti-MEM (Thermo Fisher Scientific, catalog no. 11058-021), and 158.24 μl 7.5 mM polyethylenimine (PEI). After ∼6 h, the medium was changed to complete PEL RPMI medium containing 20% Serum Plus II medium supplement, as detailed above. Supernatants containing lentiviruses were harvested 72 h posttransfection by passage through a 0.45-μm-pore-size filter. For other types of dishes, transfections were scaled roughly based on culture area.

### Functional titration of lentiviral vectors containing antibiotic resistance cassettes.

All lentiviral sgRNA preparations were subjected to functional titration of antibiotic resistance in naive PEL cell lines. This approach ensures that any phenotype observed upon sgRNA delivery are sgRNA specific and not due to differences in titer. For this, viral titers were determined by infecting naive PEL cell lines using serial dilutions of the viral supernatant in the presence of a final concentration of 4 μg/ml Polybrene in a 24-well plate at a concentration of 2 × 10^5^ cells/ml. After 24 h, infected cells were selected with puromycin, blasticidin, or hygromycin for 2 or 3 days, until no viable control cells remained. Titers of lentiviral cDNA vectors used for enhancer reporter assays were determined functionally on BJAB cells by the use of the corresponding antibiotics. IRF4-IRES-puro titers were read after 2 days of puromycin selection (1 μg/ml), while vIRF3-IRES-blast and BATF-IRES-zeo titers were read after 3 days of blasticidin selection (10 μg/ml) or zeocin selection (150 μg/ml).

In all cases, the percentages of cells surviving at relevant dilutions were used to calculate titers (in transducing units per milliliter) using the Poisson distribution.

### RNA-based titration of enhancer reporter lentiviruses.

Since the reporter lentiviruses do not carry resistance genes for antibiotic selection in mammalian cells and since only the SV40-eGFP/mCherry constructs were amenable to functional titration by fluorescence-activated cell sorter (FACS) analysis in naive cells, viral RNAs were purified directly from 200 μl of filtered lentiviral supernatant using a GeneJET viral DNA and RNA purification kit (Thermo Scientific) and eluted in 50 μl H_2_O. Absolute genome copy numbers were determined by quantitative PCR (qPCR), based on serial dilutions of purified viral RNA, using a Lenti-X quantitative real-time PCR (qRT-PCR) titration kit (Clontech) and a LightCycler 480 system (Roche). In parallel, titers of SV40-eGFP and SV40-mCherry lentiviruses were determined functionally by FACS analysis, 2 days after transduction of BJAB cells. The functional titers of the mP and DUSP22 reporter viruses were then calculated based on the ratios of the genome copy numbers from the qRT-PCR and the SV40-eGFP FACS-based titers.

### Inducible Cas9 BCBL-1.

Lentivirus expressing a doxycycline (Dox)-inducible Cas9 was produced from pCW-Cas9 (a gift from Eric Lander and David Sabatini; Addgene catalog no. 50661) and transduced into BCBL-1 cells at MOI of <1.5. After puromycin selection, single-cell clones were derived using dilution cloning into round-bottom 96-well plates. Individual cell clones were initially screened for little to no basal expression of Cas9 in the absence of Dox, high expression of Cas9 upon the addition of Dox, and their ability to still undergo lytic reactivation upon treatment with 20 ng/ml 12-O-tetradecanoylphorbol 13-acetate (TPA), using Western blotting for the viral lytic genes RTA and K-bZIP as a readout of lytic reactivation. A subset of cell clones satisfying these conditions was then functionally screened for efficient gene editing using pLX-sgRNA (encoding sgAAVS1) as a negative control and sgIRF4 as a positive control for an essential gene, since we have previously established IRF4 as critical in BCBL-1. BCBL-1/CwCas9 clone 12 was chosen for this study. To generate the Dox-inducible BCBL-1/CwCas9-sgRNA cell lines used in this study, BCBL-1 CwCas9 clone 12 was transduced with lentiviruses from pLX vectors expressing sgRNAs against the intergenic AAVS1 locus (pLX-sgRNA) or IRF4 or BATF coding regions at an MOI of ∼1. sgRNA-transduced cell lines were selected with blasticidin, in the absence of Dox, thereby preventing gene editing. Efficient editing upon Dox-induced expression of Cas9 was confirmed in pilot experiments, using Western blotting for IRF4 or BATF.

### CRISPRi.

dCas9-KRAB cells were made by infecting early passage BC-1 or BC-3 cells with a lentiviral preparation generated using pMK1316 (a gift from Martin Kampmann) at an MOI of 1.5. pMK1316 coexpresses the dCas9-KRAB fusion protein with an mCherry reporter using a P2A ribosomal skipping peptide, transcribed from the human ubiquitin C promoter. Cells expressing high levels of mCherry (top 20% of total mCherry^+^ population) were sorted at 4°C on a BD FacsMelody or FacsAria cell sorter (Robert H. Lurie Comprehensive Cancer Center Flow Cytometry Core Facility). CRISPRi competence of dCas9-KRAB cells was assessed by growth curve analysis and IRF4 Western blotting upon lentiviral transduction of the IRF4 sg-i1 sgRNA from the CRISPRi Dolcetto library (75).

### Infection of PEL cell lines with enhancer reporters.

BC-3, BC-1, or BCBL-1 cells (5 × 10^5^) were plated in wells of a 6-well plate and transduced with mP-, SV40-, or IRF4-SE-eGFP lentiviruses (MOI 2) in the presence of 2 μl of Polybrene (10 μg/μl). After 4 days, eGFP fluorescence was measured by FACS analysis on a BD FACSCelesta flow cytometer (BD Biosciences) and analyzed using FlowJo v10.6.2 (BD Biosciences).

### Enhancer reporter assay.

HEK293 cells (5 × 10^4^ cells per assay) were transduced with enhancer reporter lentiviruses and combinations of cDNA lentiviruses (at MOI 3 for each virus) at the time of plating in 24-well plates. After 3 days, cells were trypsinized and subjected to FACS analysis. For the Western blots shown in Fig. 5C, HEK293 cells were infected as described above but scaled to a 6-well format (2.4 × 10^5^ cells per sample). After 3 days, cells were scraped into 1 ml phosphate-buffered saline (PBS; 137 mM NaCl, 2.7 mM KCl, 1.8 mM KH_2_PO_4_, 10 mM Na_2_HPO_4_, pH 7.4), collected by low-speed centrifugation, lysed with 60 μl complete radioimmunoprecipitation assay (RIPA) buffer, and sonicated for 5 min on the high setting (30 s on, 30 s off), as outlined below.

### Immunoprecipitation.

Endogenous coimmunoprecipitations (co-IP) of IRF4 were performed using 10^7^ BC-3 cells per reaction mixture and a nuclear complex co-IP kit (Active Motif, Carlsbad, CA, catalog no. 54001), following the manufacturer’s recommended protocol. Briefly, 90% of the nuclear extract from ∼10^7^ BC3 cells was used for immunoprecipitation with 500 ng of anti-IRF4 antibody (Cell Signaling Technologies, catalog no. D9P5H) or rabbit IgG isotype control in high-stringency IP buffer supplemented with an additional 150 mM NaCl. Antibody complexes were recovered using Dynabeads protein A (Thermo Fisher Scientific, catalog no. 10001D). After stringent washing performed using the nuclear complex co-IP kit as instructed, magnetic beads were resuspended in wash buffer with a final concentration of 1× lithium dodecyl sulfate loading buffer. One-quarter of the immunoprecipitate was run to probe for IRF4, while three-quarters were loaded to probe for coimmunoprecipitated vIRF3 and analyzed by quantitative Western blotting using a Li-Cor platform. To estimate association of vIRF3 with IRF4, the percentage of coprecipitated vIRF3 was normalized to the efficiency of IRF4 immunoprecipitation. Under the conditions used, IRF4 coprecipitated 15.2%, 48.5%, and 16.6% of input vIRF3 in three biological replicates of the experiments.

For co-IP using tagged cDNA, HEK293T cells were transfected with the various indicated combinations of plasmid vectors encoding epitope/affinity-tagged vIRF-3 (pCEBZ-vIRF3-SF) (70), IRF4 (pCEBZ-IRF4-CBD), or VKORCv1 (pCEBZ-VKORC1v1-CBD [74]), using 2.5 μg of each vector per 10-cm-diameter dish along with 12.5 μg/ml PEI. DNA- and PEI-containing media were replaced with fresh medium after 8 h, and the cells were incubated for a further 48 h before harvest. Cell monolayers were rinsed with PBS, harvested into 1 ml of PBS, pelleted by low-speed centrifugation, and resuspended in 1 ml of ice-cold lysis buffer (50 mM Tris, 150 mM NaCl, 5 mM EDTA, 0.2% NP-40, pH 7.4) containing 1% protease inhibitor cocktail (Sigma-Aldrich, catalog no. P8340) prior to cell disruption by sonication (20 s, microtip sonicator). Cell extracts were cleared by centrifugation at 12,000 × *g* for 15 min, and supernatants were divided for incubation overnight at 4°C with either anti-FLAG M2 magnetic beads (Sigma, catalog no. 8823) or chitin resin beads (New England BioLabs, catalog no. S6651L). Beads and bound proteins were washed in lysis buffer by repeated centrifugation sedimentation and resuspension (×4) prior to final resuspension in denaturing gel loading buffer (100 mM Tris, 4% SDS, 200 mM dithiothreitol [DTT], 0.2% bromophenol blue, 20% glycerol, pH 6.8).

### Growth curves.

Cas9 or dCas9-KRAB modified PEL cell lines were infected with lentiviruses encoding sgRNAs at MOI 1 in the presence of 4 μg/ml Polybrene at a cell density of 2 × 10^5^ cells/ml. After 24 h, supernatant was replaced with fresh complete medium and 1 μg/ml puromycin. Live-cell counts were determined daily using trypan blue exclusion assay. Cell concentrations were adjusted to 2 × 10^5^ cells/ml when cell populations reached a density of at least 7 × 10^5^ cells/ml. From the dilution factors, cumulative live-cell counts were calculated. Growth curve analyses for KO or KD cells were terminated when live-cell counts decreased below 10% of sgAAVS1 control cells. Growth curves were plotted as a percentage of the cumulative live-cell count of sgAAVS1 control.

### Western blotting.

Samples from co-IP experiments in HEK293T cells (Fig. S4) were resolved on 10% polyacrylamide gels containing 0.1% SDS and transferred electrophoretically to nitrocellulose membranes. These were incubated overnight at 4°C in TBST (Tris-buffered saline with Tween 20; 20 mM Tris, 137 mM NaCl, 0.1% Tween 20, pH 7.5) containing 5% dissolved skim milk powder and primary antibodies (see Table S7). Membranes were then rinsed with TBST and incubated with horseradish peroxidase (HRP)-conjugated anti-mouse secondary antibodies (1:10,000 in TBST containing 5% dissolved skim milk powder) for 1 h at room temperature before chemiluminescence imaging (ECL; GE Healthcare, catalog no. RPN2106).

For other protein samples, cells were lysed in ice-cold RIPA buffer (25 mM Tris, 150 mM NaCl, 1% IGEPAL-CA-630, 0.5% sodium deoxycholate, 0.1% sodium dodecyl sulfate, pH 7.4) supplemented with 1× protease inhibitor cocktail III (Calbiochem, EMD Millipore). Lysates were sonicated for 5 to 7 cycles (30 s on, 30 s off) at 4°C using the highest setting of a Bioruptor sonication system (Diagenode). Cell debris was removed by centrifugation at 14,000 × *g* for 10 min at 4°C. Cleared supernatants were collected and quantified using a bicinchoninic acid (BCA) protein assay kit (Thermo Fisher). Equivalent amounts of total protein (10 to 30 μg) were resolved in Bolt 4%-to-12% Bis-Tris gradient gels (Thermo Fisher Scientific) and transferred to 0.22-μm-pore-size nitrocellulose membranes. Immunoblots were blocked with a 1:3 dilution of Odyssey blocking buffer (Li-Cor Biosciences, catalog no. 927-40000)–PBS for 1 h at room temperature. Membranes were incubated with primary antibodies (see Table S7) in blocking buffer–0.1% Tween 20 overnight at 4°C. Immunoblots were washed five times with TBST for 8 min each time and incubated with IRDye 800 CW-conjugated secondary antibodies (Li-Cor Biosciences) (1:10,000) mixed with TBS or HRP-conjugated secondary antibodies (Cell Signaling) (1:30,000) mixed with TBST and 5% dissolved skim milk powder. Membranes were washed five times with TBST, rinsed with TBS, and imaged with an Odyssey Fc dual-mode imaging system (Li-Cor Biosciences) in the infrared channel with a 785-nm-wavelength laser or in the chemiluminescent channel after incubation in SuperSignal West Femto maximum-sensitivity substrate for 5 min (Thermo Fisher Scientific, catalog no. 34095).

### Immunofluorescence.

For immunofluorescence analyses, BC-3/dCas9-KRAB cells were transduced with the specified sgRNAs at MOI 1. Control cells were treated with dimethyl sulfoxide (DMSO) or 20 ng/ml TPA 1 day later. At 3 days (for CRISPRi) or 2 days (for DMSO/TPA controls) after transduction or treatment, respectively, cells were collected by low-speed centrifugation, washed with PBS, and resuspended in PBS at 60,000 cells/20 μl. Aliquots (20 μl) of each sample were spotted onto glass coverslips and air dried at 37°C until no liquid remained, typically for 10 to 15 min. Coverslips were fixed for 15 min with 4% formaldehyde–PBS, rinsed three times with PBS, permeabilized for 15 min in PBS containing 0.1% Triton X-100, rinsed three times with PBS, and blocked for 45 min in PBS containing 2% bovine serum albumin (BSA) fraction V. Samples were again rinsed three times with PBS, incubated with anti-K-bZIP primary antibody diluted 1:200 into PBS containing 0.5% BSA for 1 h at 37°C in a humidified chamber, rinsed three times, and incubated with goat anti-mouse IgG (H+L) cross-adsorbed secondary antibody conjugated to Alexa Fluor 647 (Thermo Fisher Scientific, catalog no. A21235) diluted 1:200 in PBS containing 0.5% BSA for 1 h at 37°C, in a humidified chamber. Finally, coverslips were rinsed three times with PBS, mounted onto glass slides using ProLong Gold antifade mountant with DAPI (4′,6-diamidino-2-phenylindole) (Thermo Fisher Scientific, catalog no. P36935), and incubated at room temperature for 24 h in the dark. Images were acquired on an EVOS FL digital inverted microscope, at ×10 magnification, using the same settings for all samples. Nuclei were counted automatically, after the NIH ImageJ “watershed” function was used to separate nuclei in close proximity, by the “Analyze Particles” function. The automated protocol was initially validated by comparison of results with manual counting of nuclei using the NIH ImageJ Cell Counter plugin. K-bZIP-positive cells were counted manually using the NIH ImageJ Cell Counter plugin. Estimates for reactivation given in the text are based on analysis of >2,000 cells in each case. Images shown are representative of two biological replicates.

### mRNA-Seq.

BC-3 Cas9 cells were infected in three technical replicates with lentiviral sgRNAs targeting AAVS1, IRF4, or BATF in the presence of 4 μg/ml Polybrene at MOI 3 at a cell density of 5 × 10^5^ cells/ml in a 100-mm-diameter dish (10 ml total/replicate). After 24 h, media were discarded and cells were washed with PBS to remove leftover viral supernatant and Polybrene and resuspended in complete RPMI 1460 media and 1 μg/ml puromycin. Cells were harvested 3 days (all samples) and 8 days (only sgAAVS1 and sgBATF) after transduction. BC-3 dCas9-KRAB cells were similarly infected and selected as described above, but with sgRNAs targeting AAVS1 or vIRF3 at MOI 3, at a cell density of 3 × 10^5^ cells/ml in a T25 flask (4 ml total). Cells were harvested 4 days after transduction. In each case, cell pellets were lysed in 1 ml TRIzol reagent (Thermo Fisher Scientific). Total RNA was extracted using a Direct-zol RNA Miniprep kit with on-column DNase I digestion. Total RNA was eluted in 50 μl water. RNA quantity and quality were assessed by standard *A*_260_/*A*_280_ measurements as well as RNA integrity number determinations by running the samples on an Agilent Bioanalyzer RNA 6000 Nano chip. RNA samples were submitted to the University of Chicago Genomics Facility for mRNA library preparation [oligo(dT) selection] and sequenced on an Illumina HiSeq 4000 sequencer with 50-bp single-end reads.

### Chromatin-immunoprecipitation (ChIP).

ChIP was performed as described previously (76). Briefly, cells were cross-linked with 1% formaldehyde–PBS for 10 min and quenched with 0.125 M glycine for 5 min at room temperature. All buffers were supplemented with 1× protease inhibitor cocktail (Roche) and 1 mM phenylmethylsulfonyl fluoride. Cells were incubated in 1 ml buffer 1 (50 mM HEPES-KOH, 140 mM NaCl, 1 mM EDTA, 10% glycerol, 0.5% NP-40, 0.25% Triton X-100) for 10 min on ice. Nuclei were then pelleted at 1,350 × *g* for 5 min and washed in 1 ml buffer 2 (10 mM Tris-HCl, 200 mM NaCl, 1 mM EDTA, 0.5 mM EGTA). Pelleted nuclei were resuspended and lysed in 1 ml buffer 3 (1% SDS, 10 mM EDTA, 50 mM Tris-HCl) by pipetting. Chromatin was sheared by sonication using a Bioruptor (Diagenode) for 15 cycles (30 s on, 30 s off, using the high setting) at 4°C. A 100-μl volume of 10% Triton X-100 was added to each sample, samples were mixed, and cell debris was removed by centrifugation at 20,000 × *g* and 4°C, for 10 min.

For each ChIP, chromatin from 1 × 10^6^ cells was diluted 1:10 in ChIP dilution buffer (0.01% SDS, 1.1% Triton X-100, 1.2 mM EDTA, 16.7 mM Tris-HCl, 167 mM NaCl) and incubated with 4 μg of the respective antibody for 16 h with rotating at 4°C. Chromatin immunocomplexes were bound to 50 μl BSA-blocked Pierce ChIP-grade protein A/G magnetic beads (Thermo Fisher Scientific, catalog no. 26162) for 1 h at 4°C. Beads were washed using increasing salt concentrations, i.e., low-salt buffer (0.1% SDS, 1% Triton X-100, 2 mM EDTA, 20 mM Tris-HCl, 150 mM NaCl), high-salt buffer (0.1% SDS, 1% Triton X-100, 2 mM EDTA, 20 mM Tris-HCl, 500 mM NaCl), and LiCl-wash buffer (0.25 M LiCl, 1% Nonidet P-40, 1% sodium deoxycholate, 1 mM EDTA, 10 mM Tris-HCl), with the final two washes performed with Tris-EDTA (TE) buffer without protease inhibitors.

Chromatin was eluted in 210 μl elution buffer (50 mM Tris-HCl [pH 8.0], 10 mM EDTA, 1% SDS) for 30 min at 65°C. De-cross-linking was performed by addition of 8 μl 5 M NaCl at 65°C overnight. Chromatin was diluted with 200 μl TE buffer and treated with 8 μl RNase A (10 mg/ml) for 2 h at 37°C. After the addition of 7 μl CaCl_2_ solution (300 mM CaCl_2_ in 10 mM Tris-HCl), the remaining protein was degraded using 4 μl proteinase K (40 mg/ml) for 1 h at 55°C. DNA was purified by standard phenol-chloroform extraction followed by ethanol precipitation. Samples were resuspended in 55 μl 10 mM Tris-HCl. Input DNA from 2.5 × 10^5^ cells was de-cross-linked and purified similarly to the ChIP samples.

Prior to library preparation, the quality of the purified DNA preparations was assessed on an Agilent Bioanalyzer high-sensitivity DNA chip. Sequencing libraries were generated from 2 to 10 ng ChIP DNA or input using a NEXTflex Illumina ChIP-Seq library prep kit (Bioo Scientific, catalog no. 5143-02) per the manufacturer´s instructions. All ChIP-Seq libraries were sequenced on an Illumina NextSeq 500 sequencer using single-read (1-by-75) flow cells or on a HiSeq 2500 system with single-read (1-by-50) flow cells. Sequencing yielded approximately 2 × 10^7^ sequencing reads per sample.

### mRNA-Seq and ChIP-Seq analysis.

mRNA-Seq reads were aligned to hg19 assembly and the KSHV BAC16 reference genome (GQ994935.1 [77]) for viral gene annotation, using a Spliced Transcripts Alignment to a Reference (STAR) aligner (78). Analyses of differential gene expression levels between gene KO/KD compared to AAVS1 controls were performed using DESeq2 (79). Analysis of ChIP-Seq data was performed using Ceto (https://github.com/ebartom/NGSbartom), an in-house modular pipeline used to analyze next-generation sequencing data through the Northwestern University (NU) Quest High Performance Computing Cluster. For data from GM12878, the following publicly available ChIP-Seq data sets were downloaded from NCBI: IRF4 (SRR351616), BATF (SRR351894), EBNA3C (SRR1035617), and input DNA (SRR351535) and H3K27ac (SRR2000432) and input DNA (SRR1784182). For data from ST1, the following ChIP-Seq data sets were obtained from NCBI: IRF4 (SRR5241422), BATF3 (SRR5241428), HBZ^BirA^* (SRR5241432), H3K27ac (SRR5241437), and IgG control IP (SRR5241421). ChIP-Seq data for multiple myeloma were also downloaded from NCBI: H3K27ac (ERR2570860) and input DNA (ERR2570861) for primary tumor from MM5; IRF4 (ERR2570835), FLI1 (ERR2570837), H3K27ac (ERR2570817), and input DNA (ERR2570818) for KMS12-BM; and H3K27ac (ERR2570840) and input DNA (ERR2570841) for primary memory B cell. All ChIP-Seq reads were aligned to hg19 or the KSHV BAC16 reference genome using Bowtie v1.1.2. Peaks were called using MACS v1.4.2. ChIP-Seq heat maps in Fig. S7A were generated using ngsplot v2.47.

### HOMER motif analysis.

Motif enrichment analyses of ChIP-Seq peaks from MACS or stitched enhancers from ROSE were performed using the default settings of the findmotifsGenome.pl function in HOMER v4.10.

### Identification of super-enhancers and typical enhancers using ROSE.

SEs and typical enhancers were identified with the program Rank Ordering of Super-enhancers (ROSE [32, 33]) using the H3K27ac ChIP-Seq peak files from MACS and BAM files aligned to hg19 for H3K27ac and matched inputs for each cell line. ROSE stitches together or combines clustered peaks to identify enhancer regions using the default distance of 12.5 kb and the TSS exclusion zone size of 2.5 kb and then calculates the enrichment of H3K27ac signal over background. Using these H3K27ac signals, enhancer regions are sorted and a best-fit curve is calculated. ROSE distinguishes SEs from typical enhancers using the slope of the tangent line to the best-fit curve (>1). SE coordinates were annotated using the annotatePeaks.pl function of HOMER.

### Data availability.

Ceto scripts are available in https://github.com/ebartom/NGSbartom. mRNA-Seq data were deposited under GSE132707 and ChIP-Seq data under GSE132777 and GSE135740. All primers and DNA sequences used for cloning can be found in Table S7 on Mendeley at https://doi.org/10.17632/9h4xyfnvny.1.

## References

[1] Chang Y, Cesarman E, Pessin MS, Lee F, Culpepper J, Knowles DM, Moore PS 1994 Identification of herpesvirus-like DNA sequences in AIDS-associated Kaposi's sarcoma. Science 266:1865–1869. doi:10.1126/science.7997879. 7997879

[2] Cesarman E, Chang Y, Moore PS, Said JW, Knowles DM 1995 Kaposi's sarcoma-associated herpesvirus-like DNA sequences in AIDS-related body-cavity-based lymphomas. N Engl J Med 332:1186–1191. doi:10.1056/NEJM199505043321802. 7700311

[3] Nador RG, Cesarman E, Chadburn A, Dawson DB, Ansari MQ, Sald J, Knowles DM 1996 Primary effusion lymphoma: a distinct clinicopathologic entity associated with the Kaposi's sarcoma-associated herpes virus. Blood 88:645–656. doi:10.1182/blood.V88.2.645.bloodjournal882645. 8695812

[4] Soulier J, Grollet L, Oksenhendler E, Cacoub P, Cazals-Hatem D, Babinet P, d'Agay MF, Clauvel JP, Raphael M, Degos L 1995 Kaposi's sarcoma-associated herpesvirus-like DNA sequences in multicentric Castleman's disease. Blood 86:1276–1280. doi:10.1182/blood.V86.4.1276.bloodjournal8641276. 7632932

[5] Arora N, Gupta A, Sadeghi N 2017 Primary effusion lymphoma: current concepts and management. Curr Opin Pulm Med 23:365–370. doi:10.1097/MCP.0000000000000384. 28399009

[6] Boulanger E, Gerard L, Gabarre J, Molina JM, Rapp C, Abino JF, Cadranel J, Chevret S, Oksenhendler E 2005 Prognostic factors and outcome of human herpesvirus 8-associated primary effusion lymphoma in patients with AIDS. J Clin Oncol 23:4372–4380. doi:10.1200/JCO.2005.07.084. 15994147

[7] Okada S, Goto H, Yotsumoto M 2014 Current status of treatment for primary effusion lymphoma. Intractable Rare Dis Res 3:65–74. doi:10.5582/irdr.2014.01010. 25364646 PMC4214239

[8] Lurain K, Polizzotto MN, Aleman K, Bhutani M, Wyvill KM, Goncalves PH, Ramaswami R, Marshall VA, Miley W, Steinberg SM, Little RF, Wilson W, Filie AC, Pittaluga S, Jaffe ES, Whitby D, Yarchoan R, Uldrick TS 2019 Viral, immunologic, and clinical features of primary effusion lymphoma. Blood 133:1753–1761. doi:10.1182/blood-2019-01-893339. 30782610 PMC6473499

[9] Hanahan D, Weinberg RA 2011 Hallmarks of cancer: the next generation. Cell 144:646–674. doi:10.1016/j.cell.2011.02.013. 21376230

[10] Cesarman E 2014 Gammaherpesviruses and lymphoproliferative disorders. Annu Rev Pathol 9:349–372. doi:10.1146/annurev-pathol-012513-104656. 24111911

[11] Wies E, Mori Y, Hahn A, Kremmer E, Sturzl M, Fleckenstein B, Neipel F 2008 The viral interferon-regulatory factor-3 is required for the survival of KSHV-infected primary effusion lymphoma cells. Blood 111:320–327. doi:10.1182/blood-2007-05-092288. 17890449

[12] Godfrey A, Anderson J, Papanastasiou A, Takeuchi Y, Boshoff C 2005 Inhibiting primary effusion lymphoma by lentiviral vectors encoding short hairpin RNA. Blood 105:2510–2518. doi:10.1182/blood-2004-08-3052. 15572586

[13] Guasparri I, Keller SA, Cesarman E 2004 KSHV vFLIP is essential for the survival of infected lymphoma cells. J Exp Med 199:993–1003. doi:10.1084/jem.20031467. 15067035 PMC2211879

[14] Horenstein MG, Nador RG, Chadburn A, Hyjek EM, Inghirami G, Knowles DM, Cesarman E 1997 Epstein-Barr virus latent gene expression in primary effusion lymphomas containing Kaposi's sarcoma-associated herpesvirus/human herpesvirus-8. Blood 90:1186–1191. doi:10.1182/blood.V90.3.1186.1186_1186_1191. 9242551

[15] Trivedi P, Takazawa K, Zompetta C, Cuomo L, Anastasiadou E, Carbone A, Uccini S, Belardelli F, Takada K, Frati L, Faggioni A 2004 Infection of HHV-8+ primary effusion lymphoma cells with a recombinant Epstein-Barr virus leads to restricted EBV latency, altered phenotype, and increased tumorigenicity without affecting TCL1 expression. Blood 103:313–316. doi:10.1182/blood-2003-05-1710. 12969959

[16] Mack AA, Sugden B 2008 EBV is necessary for proliferation of dually infected primary effusion lymphoma cells. Cancer Res 68:6963–6968. doi:10.1158/0008-5472.CAN-08-0627. 18757410 PMC2587434

[17] McHugh D, Caduff N, Barros MHM, Rämer PC, Raykova A, Murer A, Landtwing V, Quast I, Styles CT, Spohn M, Fowotade A, Delecluse H-J, Papoudou-Bai A, Lee Y-M, Kim J-M, Middeldorp J, Schulz TF, Cesarman E, Zbinden A, Capaul R, White RE, Allday MJ, Niedobitek G, Blackbourn DJ, Grundhoff A, Münz C 2017 Persistent KSHV Infection increases EBV-associated tumor formation in vivo via enhanced EBV lytic gene expression. Cell Host Microbe 22:61–73.e7. doi:10.1016/j.chom.2017.06.009. 28704654

[18] Bigi R, Landis JT, An H, Caro-Vegas C, Raab-Traub N, Dittmer DP 2018 Epstein-Barr virus enhances genome maintenance of Kaposi sarcoma-associated herpesvirus. Proc Natl Acad Sci U S A 115:E11379–E11387. doi:10.1073/pnas.1810128115. 30429324 PMC6275488

[19] Petre CE, Sin SH, Dittmer DP 2007 Functional p53 signaling in Kaposi's sarcoma-associated herpesvirus lymphomas: implications for therapy. J Virol 81:1912–1922. doi:10.1128/JVI.01757-06. 17121789 PMC1797584

[20] Boulanger E, Marchio A, Hong SS, Pineau P 2009 Mutational analysis of TP53, PTEN, PIK3CA and CTNNB1/beta-catenin genes in human herpesvirus 8-associated primary effusion lymphoma. Haematologica 94:1170–1174. doi:10.3324/haematol.2009.007260. 19608668 PMC2719041

[21] Manzano M, Patil A, Waldrop A, Dave SS, Behdad A, Gottwein E 2018 Gene essentiality landscape and druggable oncogenic dependencies in herpesviral primary effusion lymphoma. Nat Commun 9:3263. doi:10.1038/s41467-018-05506-9. 30111820 PMC6093911

[22] Shaffer AL, Emre NC, Lamy L, Ngo VN, Wright G, Xiao W, Powell J, Dave S, Yu X, Zhao H, Zeng Y, Chen B, Epstein J, Staudt LM 2008 IRF4 addiction in multiple myeloma. Nature 454:226–231. doi:10.1038/nature07064. 18568025 PMC2542904

[23] Yang Y, Shaffer AL III, Emre NC, Ceribelli M, Zhang M, Wright G, Xiao W, Powell J, Platig J, Kohlhammer H, Young RM, Zhao H, Yang Y, Xu W, Buggy JJ, Balasubramanian S, Mathews LA, Shinn P, Guha R, Ferrer M, Thomas C, Waldmann TA, Staudt LM 2012 Exploiting synthetic lethality for the therapy of ABC diffuse large B cell lymphoma. Cancer Cell 21:723–737. doi:10.1016/j.ccr.2012.05.024. 22698399 PMC4059833

[24] Care MA, Cocco M, Laye JP, Barnes N, Huang Y, Wang M, Barrans S, Du M, Jack A, Westhead DR, Doody GM, Tooze RM 2014 SPIB and BATF provide alternate determinants of IRF4 occupancy in diffuse large B-cell lymphoma linked to disease heterogeneity. Nucleic Acids Res 42:7591–7610. doi:10.1093/nar/gku451. 24875472 PMC4081075

[25] Schleussner N, Merkel O, Costanza M, Liang HC, Hummel F, Romagnani C, Durek P, Anagnostopoulos I, Hummel M, Johrens K, Niedobitek A, Griffin PR, Piva R, Sczakiel HL, Woessmann W, Damm-Welk C, Hinze C, Stoiber D, Gillissen B, Turner SD, Kaergel E, von Hoff L, Grau M, Lenz G, Dorken B, Scheidereit C, Kenner L, Janz M, Mathas S 2018 The AP-1-BATF and -BATF3 module is essential for growth, survival and TH17/ILC3 skewing of anaplastic large cell lymphoma. Leukemia 32:1994–2007. doi:10.1038/s41375-018-0045-9. 29588546 PMC6127090

[26] Weilemann A, Grau M, Erdmann T, Merkel O, Sobhiafshar U, Anagnostopoulos I, Hummel M, Siegert A, Hayford C, Madle H, Wollert-Wulf B, Fichtner I, Dorken B, Dirnhofer S, Mathas S, Janz M, Emre NC, Rosenwald A, Ott G, Lenz P, Tzankov A, Lenz G 2015 Essential role of IRF4 and MYC signaling for survival of anaplastic large cell lymphoma. Blood 125:124–132. doi:10.1182/blood-2014-08-594507. 25359993

[27] Nakagawa M, Shaffer AL III, Ceribelli M, Zhang M, Wright G, Huang DW, Xiao W, Powell J, Petrus MN, Yang Y, Phelan JD, Kohlhammer H, Dubois SP, Yoo HM, Bachy E, Webster DE, Yang Y, Xu W, Yu X, Zhao H, Bryant BR, Shimono J, Ishio T, Maeda M, Green PL, Waldmann TA, Staudt LM 2018 Targeting the HTLV-I-regulated BATF3/IRF4 transcriptional network in adult T cell leukemia/lymphoma. Cancer Cell 34:286–297.e10. doi:10.1016/j.ccell.2018.06.014. 30057145 PMC8078141

[28] Kataoka K, Nagata Y, Kitanaka A, Shiraishi Y, Shimamura T, Yasunaga J-I, Totoki Y, Chiba K, Sato-Otsubo A, Nagae G, Ishii R, Muto S, Kotani S, Watatani Y, Takeda J, Sanada M, Tanaka H, Suzuki H, Sato Y, Shiozawa Y, Yoshizato T, Yoshida K, Makishima H, Iwanaga M, Ma G, Nosaka K, Hishizawa M, Itonaga H, Imaizumi Y, Munakata W, Ogasawara H, Sato T, Sasai K, Muramoto K, Penova M, Kawaguchi T, Nakamura H, Hama N, Shide K, Kubuki Y, Hidaka T, Kameda T, Nakamaki T, Ishiyama K, Miyawaki S, Yoon S-S, Tobinai K, Miyazaki Y, Takaori-Kondo A, Matsuda F, Takeuchi K, Nureki O, Aburatani H, Watanabe T, Shibata T, Matsuoka M, Miyano S, Shimoda K, Ogawa S 2015 Integrated molecular analysis of adult T cell leukemia/lymphoma. Nat Genet 47:1304–1315. doi:10.1038/ng.3415. 26437031

[29] Ma Y, Walsh MJ, Bernhardt K, Ashbaugh CW, Trudeau SJ, Ashbaugh IY, Jiang S, Jiang C, Zhao B, Root DE, Doench JG, Gewurz BE 2017 CRISPR/Cas9 screens reveal Epstein-Barr virus-transformed B cell host dependency factors. Cell Host Microbe 21:580–591.e7. doi:10.1016/j.chom.2017.04.005. 28494239 PMC8938989

[30] Xu D, Zhao L, Del Valle L, Miklossy J, Zhang L 2008 Interferon regulatory factor 4 is involved in Epstein-Barr virus-mediated transformation of human B lymphocytes. J Virol 82:6251–6258. doi:10.1128/JVI.00163-08. 18417578 PMC2447047

[31] Jin Y, Chen K, De Paepe A, Hellqvist E, Krstic AD, Metang L, Gustafsson C, Davis RE, Levy YM, Surapaneni R, Wallblom A, Nahi H, Mansson R, Lin YC 2018 Active enhancer and chromatin accessibility landscapes chart the regulatory network of primary multiple myeloma. Blood 131:2138–2150. doi:10.1182/blood-2017-09-808063. 29519805 PMC6014038

[32] Loven J, Hoke HA, Lin CY, Lau A, Orlando DA, Vakoc CR, Bradner JE, Lee TI, Young RA 2013 Selective inhibition of tumor oncogenes by disruption of super-enhancers. Cell 153:320–334. doi:10.1016/j.cell.2013.03.036. 23582323 PMC3760967

[33] Whyte WA, Orlando DA, Hnisz D, Abraham BJ, Lin CY, Kagey MH, Rahl PB, Lee TI, Young RA 2013 Master transcription factors and mediator establish super-enhancers at key cell identity genes. Cell 153:307–319. doi:10.1016/j.cell.2013.03.035. 23582322 PMC3653129

[34] Hnisz D, Abraham BJ, Lee TI, Lau A, Saint-Andre V, Sigova AA, Hoke HA, Young RA 2013 Super-enhancers in the control of cell identity and disease. Cell 155:934–947. doi:10.1016/j.cell.2013.09.053. 24119843 PMC3841062

[35] Gopalakrishnan R, Matta H, Tolani B, Triche T Jr, Chaudhary PM 2016 Immunomodulatory drugs target IKZF1-IRF4-MYC axis in primary effusion lymphoma in a cereblon-dependent manner and display synergistic cytotoxicity with BRD4 inhibitors. Oncogene 35:1797–1810. doi:10.1038/onc.2015.245. 26119939 PMC4486341

[36] Patil A, Manzano M, Gottwein E 2018 CK1alpha and IRF4 are essential and independent effectors of immunomodulatory drugs in primary effusion lymphoma. Blood 132:577–586. doi:10.1182/blood-2018-01-828418. 29954751 PMC6085990

[37] Davis DA, Mishra S, Anagho HA, Aisabor AI, Shrestha P, Wang V, Takamatsu Y, Maeda K, Mitsuya H, Zeldis JB, Yarchoan R 2017 Restoration of immune surface molecules in Kaposi sarcoma-associated herpes virus infected cells by lenalidomide and pomalidomide. Oncotarget 8:50342–50358. doi:10.18632/oncotarget.17960. 28881567 PMC5584136

[38] Tolani B, Gopalakrishnan R, Punj V, Matta H, Chaudhary PM 2014 Targeting Myc in KSHV-associated primary effusion lymphoma with BET bromodomain inhibitors. Oncogene 33:2928–2937. doi:10.1038/onc.2013.242. 23792448 PMC4892892

[39] Glasmacher E, Agrawal S, Chang AB, Murphy TL, Zeng W, Vander Lugt B, Khan AA, Ciofani M, Spooner CJ, Rutz S, Hackney J, Nurieva R, Escalante CR, Ouyang W, Littman DR, Murphy KM, Singh H 2012 A genomic regulatory element that directs assembly and function of immune-specific AP-1-IRF complexes. Science 338:975–980. doi:10.1126/science.1228309. 22983707 PMC5789805

[40] Li P, Spolski R, Liao W, Wang L, Murphy TL, Murphy KM, Leonard WJ 2012 BATF-JUN is critical for IRF4-mediated transcription in T cells. Nature 490:543–546. doi:10.1038/nature11530. 22992523 PMC3537508

[41] Murphy TL, Tussiwand R, Murphy KM 2013 Specificity through cooperation: BATF-IRF interactions control immune-regulatory networks. Nat Rev Immunol 13:499–509. doi:10.1038/nri3470. 23787991

[42] Zhou F, Shimoda M, Olney L, Lyu Y, Tran K, Jiang G, Nakano K, Davis RR, Tepper CG, Maverakis E, Campbell M, Li Y, Dandekar S, Izumiya Y 2017 Oncolytic reactivation of KSHV as a therapeutic approach for primary effusion lymphoma. Mol Cancer Ther 16:2627–2638. doi:10.1158/1535-7163.MCT-17-0041. 28847988 PMC5914504

[43] Rivas C, Thlick AE, Parravicini C, Moore PS, Chang Y 2001 Kaposi's sarcoma-associated herpesvirus LANA2 is a B-cell-specific latent viral protein that inhibits p53. J Virol 75:429–438. doi:10.1128/JVI.75.1.429-438.2001. 11119611 PMC113935

[44] Wies E, Hahn AS, Schmidt K, Viebahn C, Rohland N, Lux A, Schellhorn T, Holzer A, Jung JU, Neipel F 2009 The Kaposi's sarcoma-associated herpesvirus-encoded vIRF-3 inhibits cellular IRF-5. J Biol Chem 284:8525–8538. doi:10.1074/jbc.M809252200. 19129183 PMC2659211

[45] Lubyova B, Kellum MJ, Frisancho AJ, Pitha PM 2004 Kaposi's sarcoma-associated herpesvirus-encoded vIRF-3 stimulates the transcriptional activity of cellular IRF-3 and IRF-7. J Biol Chem 279:7643–7654. doi:10.1074/jbc.M309485200. 14668346

[46] Qi LS, Larson MH, Gilbert LA, Doudna JA, Weissman JS, Arkin AP, Lim WA 2013 Repurposing CRISPR as an RNA-guided platform for sequence-specific control of gene expression. Cell 152:1173–1183. doi:10.1016/j.cell.2013.02.022. 23452860 PMC3664290

[47] Gilbert LA, Larson MH, Morsut L, Liu Z, Brar GA, Torres SE, Stern-Ginossar N, Brandman O, Whitehead EH, Doudna JA, Lim WA, Weissman JS, Qi LS 2013 CRISPR-mediated modular RNA-guided regulation of transcription in eukaryotes. Cell 154:442–451. doi:10.1016/j.cell.2013.06.044. 23849981 PMC3770145

[48] Aguirre AJ, Meyers RM, Weir BA, Vazquez F, Zhang CZ, Ben-David U, Cook A, Ha G, Harrington WF, Doshi MB, Kost-Alimova M, Gill S, Xu H, Ali LD, Jiang G, Pantel S, Lee Y, Goodale A, Cherniack AD, Oh C, Kryukov G, Cowley GS, Garraway LA, Stegmaier K, Roberts CW, Golub TR, Meyerson M, Root DE, Tsherniak A, Hahn WC 2016 Genomic copy number dictates a gene-independent cell response to CRISPR/Cas9 targeting. Cancer Discov 6:914–929. doi:10.1158/2159-8290.CD-16-0154. 27260156 PMC4972686

[49] Munoz DM, Cassiani PJ, Li L, Billy E, Korn JM, Jones MD, Golji J, Ruddy DA, Yu K, McAllister G, DeWeck A, Abramowski D, Wan J, Shirley MD, Neshat SY, Rakiec D, de Beaumont R, Weber O, Kauffmann A, McDonald ER III, Keen N, Hofmann F, Sellers WR, Schmelzle T, Stegmeier F, Schlabach MR 2016 CRISPR screens provide a comprehensive assessment of cancer vulnerabilities but generate false-positive hits for highly amplified genomic regions. Cancer Discov 6:900–913. doi:10.1158/2159-8290.CD-16-0178. 27260157

[50] Heinz S, Benner C, Spann N, Bertolino E, Lin YC, Laslo P, Cheng JX, Murre C, Singh H, Glass CK 2010 Simple combinations of lineage-determining transcription factors prime cis-regulatory elements required for macrophage and B cell identities. Mol Cell 38:576–589. doi:10.1016/j.molcel.2010.05.004. 20513432 PMC2898526

[51] Arguello M, Sgarbanti M, Hernandez E, Mamane Y, Sharma S, Servant M, Lin R, Hiscott J 2003 Disruption of the B-cell specific transcriptional program in HHV-8 associated primary effusion lymphoma cell lines. Oncogene 22:964–973. doi:10.1038/sj.onc.1206270. 12592383

[52] Goto H, Kariya R, Kudo E, Okuno Y, Ueda K, Katano H, Okada S 2017 Restoring PU.1 induces apoptosis and modulates viral transactivation via interferon-stimulated genes in primary effusion lymphoma. Oncogene 36:5252–5262. doi:10.1038/onc.2017.138. 28481873

[53] Rickels R, Shilatifard A 2018 Enhancer logic and mechanics in development and disease. Trends Cell Biol 28:608–630. doi:10.1016/j.tcb.2018.04.003. 29759817

[54] Li X, Chen S, Feng J, Deng H, Sun R 2010 Myc is required for the maintenance of Kaposi's sarcoma-associated herpesvirus latency. J Virol 84:8945–8948. doi:10.1128/JVI.00244-10. 20573831 PMC2919007

[55] Clappier E, Cuccuini W, Kalota A, Crinquette A, Cayuela JM, Dik WA, Langerak AW, Montpellier B, Nadel B, Walrafen P, Delattre O, Aurias A, Leblanc T, Dombret H, Gewirtz AM, Baruchel A, Sigaux F, Soulier J 2007 The C-MYB locus is involved in chromosomal translocation and genomic duplications in human T-cell acute leukemia (T-ALL), the translocation defining a new T-ALL subtype in very young children. Blood 110:1251–1261. doi:10.1182/blood-2006-12-064683. 17452517

[56] Lahortiga I, De Keersmaecker K, Van Vlierberghe P, Graux C, Cauwelier B, Lambert F, Mentens N, Beverloo HB, Pieters R, Speleman F, Odero MD, Bauters M, Froyen G, Marynen P, Vandenberghe P, Wlodarska I, Meijerink JP, Cools J 2007 Duplication of the MYB oncogene in T cell acute lymphoblastic leukemia. Nat Genet 39:593–595. doi:10.1038/ng2025. 17435759

[57] Anfossi G, Gewirtz AM, Calabretta B 1989 An oligomer complementary to c-myb-encoded mRNA inhibits proliferation of human myeloid leukemia cell lines. Proc Natl Acad Sci U S A 86:3379–3383. doi:10.1073/pnas.86.9.3379. 2541445 PMC287136

[58] Calabretta B, Sims RB, Valtieri M, Caracciolo D, Szczylik C, Venturelli D, Ratajczak M, Beran M, Gewirtz AM 1991 Normal and leukemic hematopoietic cells manifest differential sensitivity to inhibitory effects of c-myb antisense oligodeoxynucleotides: an in vitro study relevant to bone marrow purging. Proc Natl Acad Sci U S A 88:2351–2355. doi:10.1073/pnas.88.6.2351. 2006173 PMC51229

[59] Hong Z, Pedersen NM, Wang L, Torgersen ML, Stenmark H, Raiborg C 2017 PtdIns3P controls mTORC1 signaling through lysosomal positioning. J Cell Biol 216:4217–4233. doi:10.1083/jcb.201611073. 29030394 PMC5716264

[60] Jiang S, Zhou H, Liang J, Gerdt C, Wang C, Ke L, Schmidt SCS, Narita Y, Ma Y, Wang S, Colson T, Gewurz B, Li G, Kieff E, Zhao B 2017 The Epstein-Barr virus regulome in lymphoblastoid cells. Cell Host Microbe 22:561–573-e4. doi:10.1016/j.chom.2017.09.001. 29024646 PMC5662195

[61] Inoue F, Kircher M, Martin B, Cooper GM, Witten DM, McManus MT, Ahituv N, Shendure J 2017 A systematic comparison reveals substantial differences in chromosomal versus episomal encoding of enhancer activity. Genome Res 27:38–52. doi:10.1101/gr.212092.116. 27831498 PMC5204343

[62] Thomas MP, Liu X, Whangbo J, McCrossan G, Sanborn KB, Basar E, Walch M, Lieberman J 2015 Apoptosis triggers specific, rapid, and global mRNA decay with 3' uridylated intermediates degraded by DIS3L2. Cell Rep 11:1079–1089. doi:10.1016/j.celrep.2015.04.026. 25959823 PMC4862650

[63] Liu X, Fu R, Pan Y, Meza-Sosa KF, Zhang Z, Lieberman J 2018 PNPT1 release from mitochondria during apoptosis triggers decay of poly(A) RNAs. Cell 174:187–201.e12. doi:10.1016/j.cell.2018.04.017. 29779946

[64] Subramanian A, Tamayo P, Mootha VK, Mukherjee S, Ebert BL, Gillette MA, Paulovich A, Pomeroy SL, Golub TR, Lander ES, Mesirov JP 2005 Gene set enrichment analysis: a knowledge-based approach for interpreting genome-wide expression profiles. Proc Natl Acad Sci U S A 102:15545–15550. doi:10.1073/pnas.0506580102. 16199517 PMC1239896

[65] Schmidt K, Wies E, Neipel F 2011 Kaposi's sarcoma-associated herpesvirus viral interferon regulatory factor 3 inhibits gamma interferon and major histocompatibility complex class II expression. J Virol 78:4530–4537. doi:10.1128/JVI.02123-10. PMC3126280 21345951

[66] Guo R, Jiang C, Zhang Y, Govande A, Trudeau SJ, Chen F, Fry CJ, Puri R, Wolinsky E, Schineller M, Frost TC, Gebre M, Zhao B, Giulino-Roth L, Doench JG, Teng M, Gewurz BE 2020 MYC controls the Epstein-Barr virus lytic switch. Mol Cell 85:653–669.e8. doi:10.1016/j.molcel.2020.03.025. PMC7245572 32315601

[67] Forero A, McCormick KD, Jenkins FJ, Sarkar SN 2014 Downregulation of IRF4 induces lytic reactivation of KSHV in primary effusion lymphoma cells. Virology 458–459:4–10. doi:10.1016/j.virol.2014.04.020. PMC4058074 24928034

[68] Wang A, Welch R, Zhao B, Ta T, Keleş S, Johannsen E 2015 Epstein-Barr virus nuclear antigen 3 (EBNA3) proteins regulate EBNA2 binding to distinct RBPJ genomic sites. J Virol 90:2906–2919. doi:10.1128/JVI.02737-15. 26719268 PMC4810642

[69] Frost TC, Gewurz BE 2018 Epigenetic crossroads of the Epstein-Barr virus B-cell relationship. Curr Opin Virol 32:15–23. doi:10.1016/j.coviro.2018.08.012. 30227386 PMC6263794

[70] Xiang Q, Ju H, Li Q, Mei SC, Chen D, Choi YB, Nicholas J 2018 Human herpesvirus 8 interferon regulatory factors 1 and 3 mediate replication and latency activities via interactions with USP7 deubiquitinase. J Virol 92:e02003-17. doi:10.1128/JVI.02003-17. 29343584 PMC5972880

[71] Doench JG, Fusi N, Sullender M, Hegde M, Vaimberg EW, Donovan KF, Smith I, Tothova Z, Wilen C, Orchard R, Virgin HW, Listgarten J, Root DE 2016 Optimized sgRNA design to maximize activity and minimize off-target effects of CRISPR-Cas9. Nat Biotechnol 34:184–191. doi:10.1038/nbt.3437. 26780180 PMC4744125

[72] Patil A, Manzano M, Gottwein E 2019 Genome-wide CRISPR screens reveal genetic mediators of cereblon modulator toxicity in primary effusion lymphoma. Blood Adv 3:2105–2117. doi:10.1182/bloodadvances.2019031732. 31300418 PMC6650732

[73] Forte E, Raja AN, Shamulailatpam P, Manzano M, Schipma MJ, Casey JL, Gottwein E 2015 MicroRNA-mediated transformation by the Kaposi's sarcoma-associated herpesvirus Kaposin locus. J Virol 89:2333–2341. doi:10.1128/JVI.03317-14. 25505059 PMC4338870

[74] Chen D, Xiang Q, Nicholas J 2017 Human Herpesvirus 8 Interleukin-6 Interacts with Calnexin Cycle Components and Promotes Protein Folding. J Virol 91:e00965-17. doi:10.1128/JVI.00965-17. 28878084 PMC5660481

[75] Sanson KR, Hanna RE, Hegde M, Donovan KF, Strand C, Sullender ME, Vaimberg EW, Goodale A, Root DE, Piccioni F, Doench JG 2018 Optimized libraries for CRISPR-Cas9 genetic screens with multiple modalities. Nat Commun 9:5416. doi:10.1038/s41467-018-07901-8. 30575746 PMC6303322

[76] Gunther T, Grundhoff A 2010 The epigenetic landscape of latent Kaposi sarcoma-associated herpesvirus genomes. PLoS Pathog 6:e1000935. doi:10.1371/journal.ppat.1000935. 20532208 PMC2880564

[77] Brulois KF, Chang H, Lee AS, Ensser A, Wong LY, Toth Z, Lee SH, Lee HR, Myoung J, Ganem D, Oh TK, Kim JF, Gao SJ, Jung JU 2012 Construction and manipulation of a new Kaposi's sarcoma-associated herpesvirus bacterial artificial chromosome clone. J Virol 6:e01019-12. doi:10.1128/JVI.01019-12. PMC3446615 22740391

[78] Dobin A, Davis CA, Schlesinger F, Drenkow J, Zaleski C, Jha S, Batut P, Chaisson M, Gingeras TR 2013 STAR: ultrafast universal RNA-seq aligner. Bioinformatics 29:15–21. doi:10.1093/bioinformatics/bts635. 23104886 PMC3530905

[79] Love MI, Huber W, Anders S 2014 Moderated estimation of fold change and dispersion for RNA-seq data with DESeq2. Genome Biol 15:550. doi:10.1186/s13059-014-0550-8. 25516281 PMC4302049

[80] Lerdrup M, Johansen JV, Agrawal-Singh S, Hansen K 2016 An interactive environment for agile analysis and visualization of ChIP-sequencing data. Nat Struct Mol Biol 23:349–357. doi:10.1038/nsmb.3180. 26926434

